# Antiproliferative Activity Screening in *Ocotea* spp. Reveals Active Compounds in *O. villosa* with a Promising Mechanism of Action against Human Breast Cancer Cell Line MCF‑7

**DOI:** 10.1021/acsomega.5c08430

**Published:** 2025-12-01

**Authors:** Fernanda Brito Leite Dornelas, Wanderleya Toledo dos Santos, Vanessa Viana Lempk, Juliana Leal Rodrigues da Costa, Clarissa Ferreira Cunha, Michael Murgu, Matheus Fernandes Alves, Albert Katchborian-Neto, José Otávio Do Amaral Corrêa, Daniela Aparecida Chagas-Paula, Fernanda Maria Pinto Vilela, Ana Cláudia Chagas de Paula

**Affiliations:** † Department of Pharmaceutical Sciences, 28113 Federal University of Juiz de Fora, Juiz de Fora, Minas Gerais 36036-900, Brazil; ‡ 424848 University Hospital, Federal University of Juiz de Fora, Juiz de Fora, Minas Gerais 36036-900, Brazil; § 36565 Waters Corporation, São Paulo, São Paulo 06455-020, Brazil; ∥ Chemistry Institute, 74347 Federal University of Alfenas, Alfenas, Minas Gerais 37130-001, Brazil; ⊥ Center of Natural Sciences and Humanities, Federal University of ABC, Santo Andre, São Paulo 09280-560, Brazil; # Department of Chemistry, Federal University of Juiz de Fora, Juiz de Fora, Minas Gerais 36036-900, Brazil

## Abstract

Breast cancer is a global public health challenge, being the most prevalent cancer and the leading cause of cancer-related mortality among women worldwide. About half of the cases occur without specific risk factors. The biological heterogeneity of the disease, including distinct molecular subtypes, variable aggressiveness, and differential treatment responses, combined with its increasing incidence, makes its management particularly complex. Furthermore, current treatments often cause severe side effects, chronic toxicity, and negatively impact patients’ quality of life. Consequently, there is an urgent need for new, safer, and more effective therapies. Natural agents are particularly capable of targeting multiple molecular pathways, which could overcome therapeutic resistance, modulate the tumor microenvironment, and induce fewer and milder adverse effects. Some species of the *Ocotea* genus have demonstrated encouraging antiproliferative activity. However, the activity against breast cancer of most species of this genus and their active compounds remains poorly investigated. Thus, this study screened 56 *Ocotea* species extracts for in vitro cytotoxic activity against MCF-7 human breast cancer cells. Among the tested species, only the crude extract of *O. villosa* leaf (CE) exhibited a significant cytotoxicity against MCF-7 (IC_50_ = 100.00 ± 6.06 μg/mL; selective index, SI = 1.3). Metabolomic profiling revealed high levels of alkaloids in CE, including chemical annotations of major aporphine compounds (e.g., nuciferine-derived alkaloids). Mechanistic studies indicated that CE has pro-apoptotic activity, inducing apoptosis of 56.59% MCF-7 population, with an activity similar to 5-FU (53.8%), inhibiting cell migration by approximately 5%, and suppressing colony formation by around 50%, without significantly changing the cell cycle. Additionally, semiquantitative RT-PCR evidenced the overexpression of the pro-apoptotic marker CASP9, suggesting intrinsic apoptosis pathway activation by CE. These findings highlight the potential of *O. villosa* as a source of promising bioactive compounds for the development of anticancer therapies. The complex composition of CE provides various chemical scaffolds as lead compounds, supporting a multitarget mechanism of action with potential in the development of more effective anticancer therapies, reducing therapy resistance and decreasing adverse effects.

## Graphical abstract

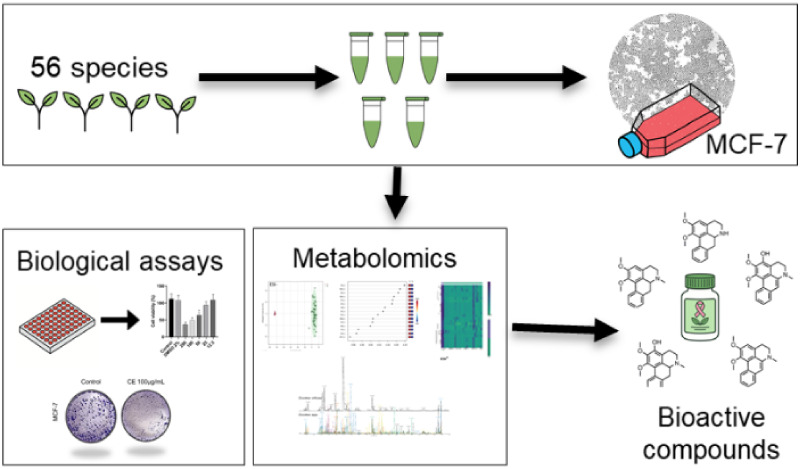

## 1 Introduction

Breast cancer remains a critical global public health challenge and is the most prevalent and deadliest cancer among women, with over 2.3 million new cases and 670,000 deaths annually as of 2022.^1,2^ Its management is complicated due to its complex etiology, and nearly half of all cases arise in the absence of specific risk factors. Its significant biological heterogeneity leads to diverse subtypes with varying aggressiveness and treatment responses.^3^ Furthermore, the rising global incidence, treatment resistance, and the severe side effects of current therapies, such as fatigue, nausea, vomiting, diarrhea, lymphedema, pain, chronic toxicity, cardiometabolic dysfunction, infertility, and cognitive deficits, which severely affect long-term patient quality of life, represent an important clinical challenge. All these facts underscore the urgent need for safer and more effective therapeutic alternatives.^4^

Breast cancer is classified histologically as ductal or lobular, and further staged from in situ (stage 0) to metastatic (stage IV).^3,4^ Molecular subtype classification is crucial for therapy selection and includes four main categories: luminal A, luminal B, HER2-positive, and basal.^3–5^ For instance, most breast tumors are estrogen receptor positive (ER+), and luminal A generally presents the best prognosis.^3,4^ Standard breast cancer therapies include surgery, chemotherapy, radiation therapy, hormone therapy, immunotherapy, and targeted drug therapy.^5,6^ Despite advances in treatment personalization, most current therapies still cause severe side effects. In response, global initiatives like the WHO’s Global Breast Cancer Initiative aim to reduce mortality by 2.5% per year until 2040 by promoting early detection and encouraging the development of innovative therapies.^1^

Natural products have historically been a source of novel anticancer agents, as evidenced by successful drugs like paclitaxel (*Taxus brevifolia*) and camptothecin (from *Camptotheca acuminata*).^7,8^ From 1981 to 2019, 35 natural products, 65 semisynthetic derivatives, and 53 fully synthetic compounds were approved for cancer treatment, illustrating the significant contribution of bioactive natural compounds to modern oncology.^9^ Natural products can act through multiple mechanisms of action, including inducing apoptosis, affecting malignant cell migration, influencing immune response, and affecting cell cycle progression, cell cycle checkpoints, and DNA damage repair machinery, among others. In addition, the anti-inflammatory, immunomodulatory, and antioxidant properties of many natural products provide a mode of action that can decrease adverse effects and overcome drug resistance.^10^

The genus *Ocotea* encompasses approximately 400 species, with several native to South America.^11,12^ It has demonstrated broad pharmacological potential.^11,12^ Several species are traditionally used for medicinal purposes, including *O. odorifera* (anti-inflammatory) and *O. quixos* (antibacterial).^12,13^ Importantly, cytotoxic effects against various cancer cell lines have been reported for *O. leucoxylon*, *O. longifolia*, and *O. acutifolia*.^14–16^ The stem extract of *O. longifolia* showed activity against Hep-G2 (human liver carcinoma) and HL60 (human leukemia) cell lines, and the leaf extract of this species was cytotoxic to Hep-G2, HL60, B16–F10 (murine melanoma), K562 (Ph+ erythroleukemia) cell lines.^15^ While there is some evidence for the genus’s therapeutic antiproliferative potential, research into its anticancer potential, particularly against breast cancer, remains limited.

In this context, metabolomic strategies, including chemical profiling, offer a powerful approach to studying the chemical diversity of natural products and identifying potential bioactive compounds within complex mixtures.^17,18^ By coupling advanced analytical techniques, such as liquid chromatography–mass spectrometry (LC-MS), with bioinformatics, these strategies enable comprehensive chemical characterization of extracts and the annotation of candidate compounds responsible for biological activity.^19,20^

Thus, in this study, we evaluated 56 *Ocotea* spp. using untargeted metabolomics and biological screening to identify extracts with antiproliferative potential. The study provides the first evidence of the antiproliferative activity of *Ocotea villosa* (Kosterm) crude leaf extract (CE) against MCF-7, an estrogen receptor-positive (ER+) breast cancer cell line, the most predominant tumor subtype. Among the 56 extracts, CE was the only one with significant cytotoxic effect. Thus, to elucidate the chemical diversity underpinning its observed effects, its mechanism of action and metabolomic information were further investigated (Figure 1). The multitarget mechanisms uncovered in this study provide a foundation for future drug discovery efforts aligned with optimized efficacy, reduced therapy resistance, and lower-toxicity alternatives to complement existing breast cancer therapies.

**Figure 1:**
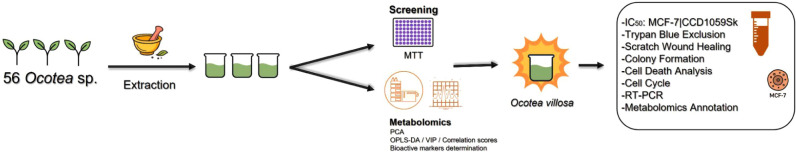
A schematic overview of the study.

## 2 Results and Discussion

### 2.1 Cell Viability Assay

The cell viability assay allowed screening of the most promising cytotoxic extracts. Extracts from 56 different *Ocotea* spp. (40 μg/mL) were evaluated, and CE was the only one that presented significant cytotoxic potential against MCF-7 cells, reducing cell viability by more than 50% (Figure 2). Thus, the CE extract was selected for further studies on its effects on MCF-7 cells, evaluating its IC_50_, selectivity, and mechanisms of action.

**Figure 2:**
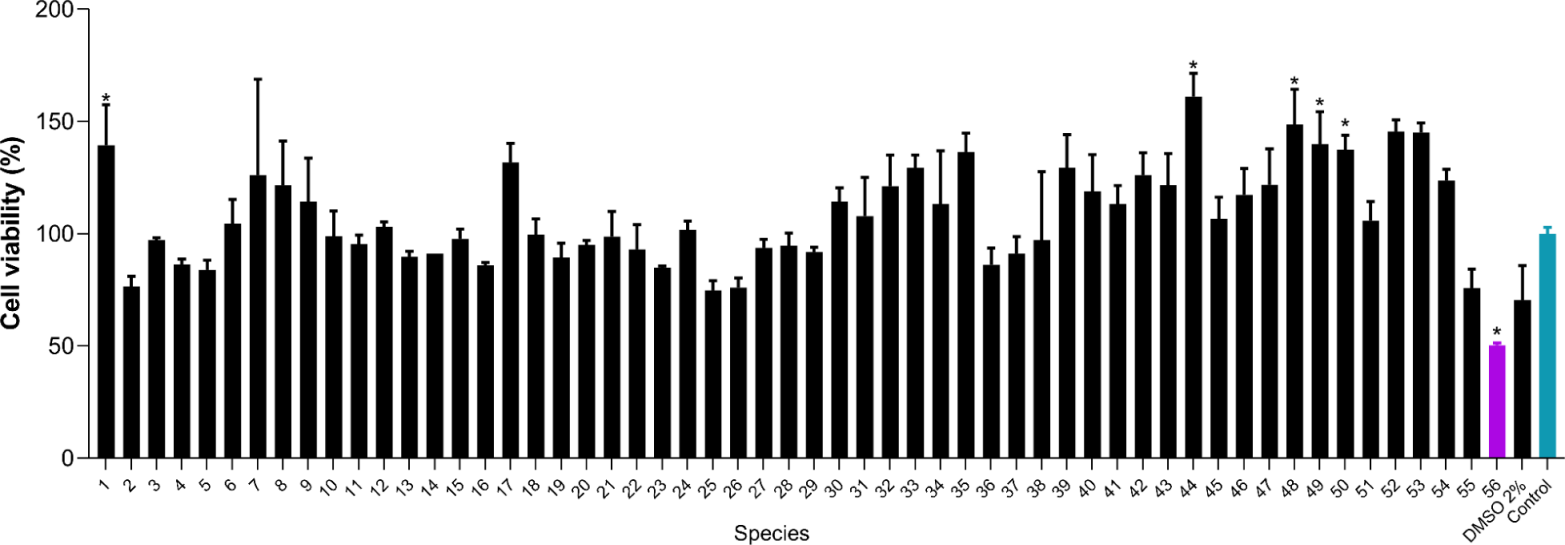
Cell viability screening determined by 3-(4,5-dimethylthiazol-2-yl)-2,5-diphenyltetrazolium bromide (MTT) assay performed 48 h after treatment of MCF-7 cells with 40 μg/mL of crude extracts of 56 *Ocotea* spp. The results are presented as mean ± SD (*n* = 3). The control group (nontreated cells) is shown in blue. The purple bar indicates the most promising extract. (*) Significant differences from the control group (untreated) were determined according to analysis of variance (ANOVA) followed by Tukey’s posttest, *p < 0.05.

Under normal conditions, epithelial and spindle-shaped morphologies were observed for MCF-7 and CCD-1059Sk cells, respectively (Figure 3A,B). Thus, the cultures were suitable for cell viability and selectivity evaluations.

**Figure 3:**
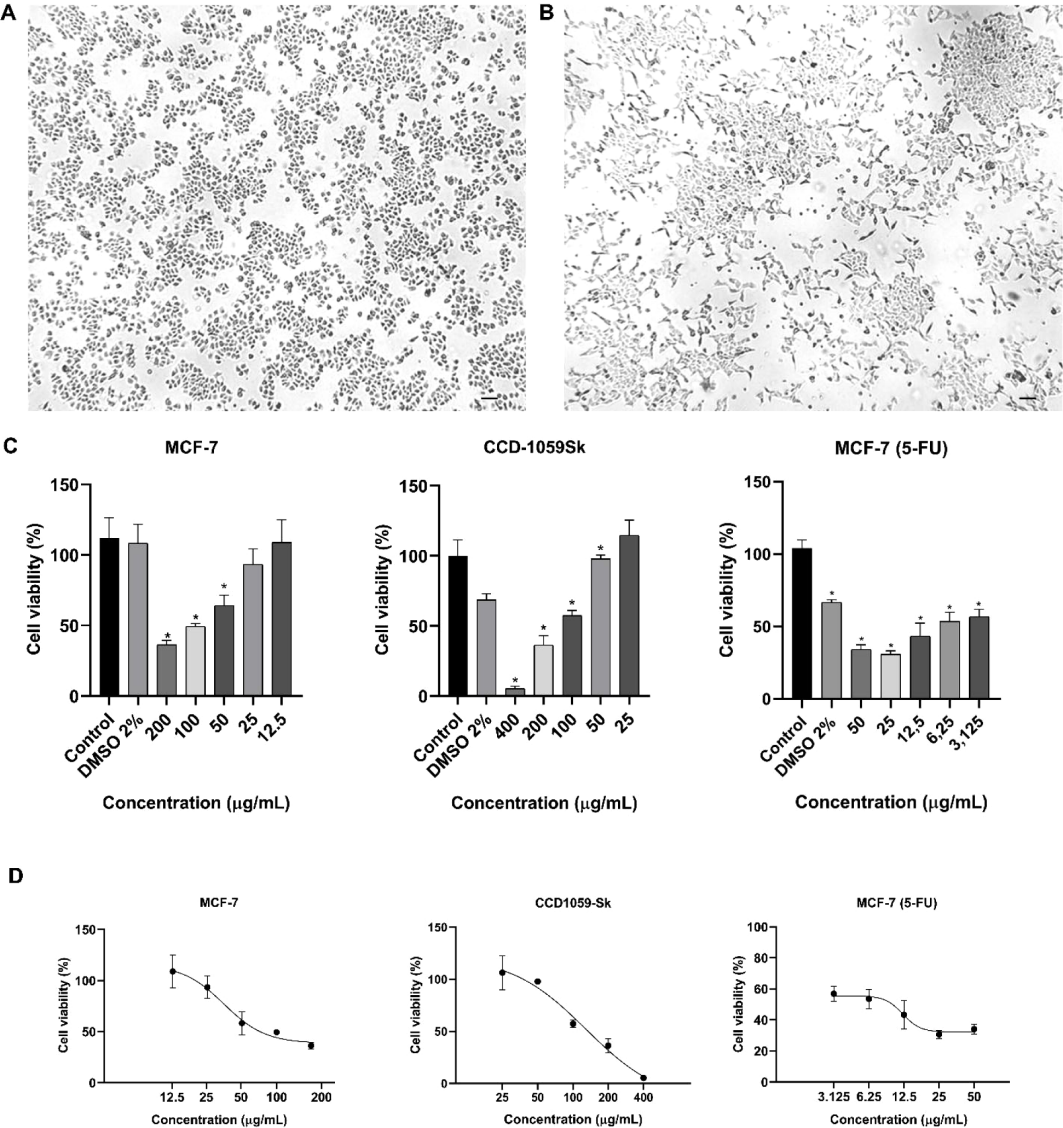
(A) Morphology of MCF-7 and (B) CCD-1059Sk cell cultures under normal conditions. (C) Cell viability evaluated for MCF-7 and CCD-1059Sk cells treated with CE and 5-FU for 48 h. (D) Dose–response curves for treatments with CE in MCF-7 and CCD-1059Sk and 5-FU in MCF-7 cells. Results are presented as mean ± SD (*n* = 3). (*) Significant differences from the control group (untreated) were determined according to analysis of variance (ANOVA) followed by Tukey’s posttest, *p < 0.05.

Regarding the viability evaluations, a significant reduction in viability was observed in the MCF-7 culture when treated with CE for 48 h with concentrations higher than 40 μg/mL. The same was not observed in the CCD-1059Sk culture treated with CE (Figure 3C). The IC_50_ values obtained for CE were 100.0 ± 3.37 μg/mL and 129.2 ± 0.88 μg/mL in MCF-7 and in CCD-1059Sk cells, respectively (Figure 3D and Table 1).

**Table 1:** IC_50_ Values (μg/ml) Determined After 48 h of Treatment with CE and 5-FU

	IC_50_ ± SD (μg/mL)	
Treatment	MCF-7	CCD-1059Sk
CE	100.0 ± 3.37	129.2 ± 0.88
5-FU	12.21 ± 1.96	–

In 2011, Garcez reported that aporphine alkaloids isolated from *O. acutifolia* presented activity against MCF-7 cells.^16^ *O. longifolia* leaf extract also exhibited cytotoxicity against Hep-G2, HL-60, B16–F10, and K562 cells, with IC_50_ values ranging from 37 to 44 μg/mL.^15^ Together, these findings support the continued investigation of *Ocotea* species, including *O. villosa*, as promising sources of bioactive compounds for cancer therapy. Moreover, no previous studies have been found in the literature evaluating CE against MCF-7 or other cancer cells.

The IC_50_ of 5-FU was 12.21 ± 1.96 μg/mL for MCF-7 (Figure 3D and Table 1), which is in agreement with values previously reported in the literature.^21^

CE showed selective activity for MCF-7 compared to the CCD-1059Sk (normal cells) with selectivity index (SI = 1.3), a result significant in the search of new treatments (Figure 3D and Table 1). Current antitumoral drugs like doxorubicin exhibit SI values against various cell lines ranging from approximately 0.57 to 1.6 (for HeLa, HCT-116, HepG2, and MCF-7), and cisplatin exhibits an SI of 0.58 against MCF-7.^22–24^ Therefore, the SI of CE is within the range observed for some gold-standard chemotherapeutic drugs and in line with the literature that considers a selective bioactive sample to present an SI > 1.^25,26^ Notwithstanding, an SI of 1.3 may be considered to represent a modest selectivity. It is worth noting that strategies can be implemented to increase the SI toward MCF-7, such as active compound isolation and development of target drug delivery systems.^27^ Moreover, combined therapy involving the combination of an extract or its active compounds with conventional chemotherapeutic drugs has emerged as a promising approach to potentiate antiproliferative effects while reducing systemic adverse effects.^28,29^

Trypan blue exclusion tests showed that cell viability decreased after 48 and 72 h in samples treated with CE at 50–150 μg/mL compared to the control group (Figure 4), corroborating the MTT results.

**Figure 4:**
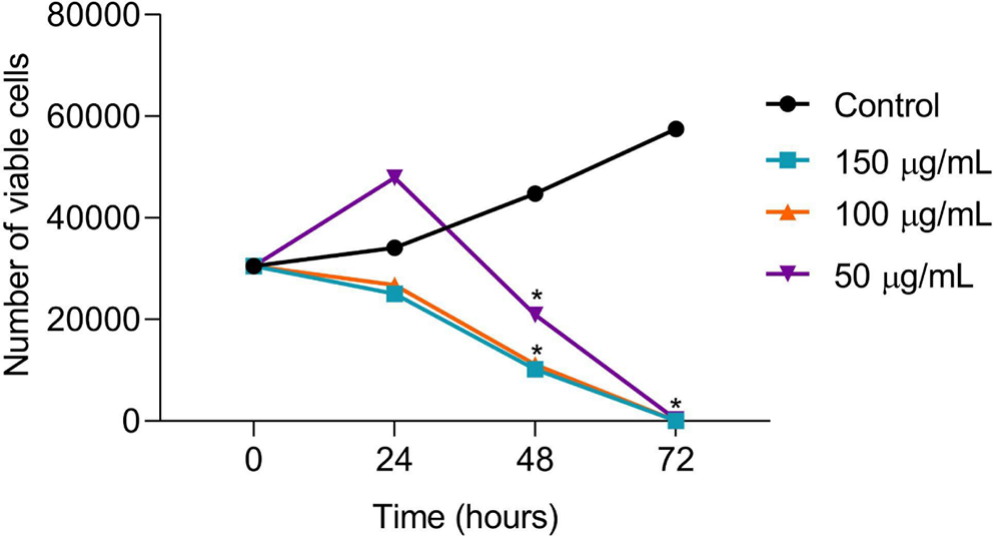
Trypan blue exclusion test showing decreasing cell proliferation after treatment with CE for 24, 48, and 72 h. Results are presented as mean ± SD (*n* = 3). Cells were treated with CE at concentrations of 50 μg/mL (purple), 100 μg/mL (orange), and 150 μg/mL (blue). The control group (nontreated cells) is shown in black. (*) Significant differences from the control group (untreated) were determined according to analysis of variance (ANOVA) followed by Tukey’s posttest, *p < 0.05.

Viability assays play a fundamental role in cancer research by providing evidence of the efficacy and selectivity of potential therapeutics.^30^ These assays enable researchers to quantify how experimental treatments affect tumor cell viability while assessing their safety in normal cells.^31^ In 2021, Normann et al. demonstrated that combining miRNAs with HER2-targeting drugs significantly reduces cell viability in HER2-positive tumor cell lines,^32^ underscoring the importance of in vitro assays for identifying compounds with therapeutic potential.^33^ Beyond measuring cell viability, these assays help uncover underlying mechanisms while guiding the selection of promising drug candidates for further studies. Their ability to efficiently and reproducibly screen compounds make them indispensable for translating preclinical discoveries into clinical applications.^32,33^ Thus, further investigation of CE is warranted.

When comparing the results obtained in this study with the data available for other *Ocotea* species, it is evident that cytotoxic activity is a recurrent feature within this genus. *O. caparrapi* exhibited growth-inhibitory activity against leukemia cells,^34^ whereas *O. leucoxylon* showed antiproliferative effects in several murine tumor cell lines, including leukemia (P-388), melanoma (B16F10), Lewis lung carcinoma (LLC), and colon cancer (Colo-205), associated with topoisomerase I inhibition.^14,35^ Extracts from *O. longifolia* demonstrated activity against multiple tumor cell lines, including HepG-2, HL-60, B16–F10, K562, and PBMC, highlighting the broad biological activity spectrum of this genus.^15,36^ In addition, *O. acutifolia* displayed cytotoxic potential against the breast cancer cell line MCF-7, attributed to the presence of the alkaloid neolitsine.^16^ Similarly, compounds isolated from *O. macrophylla*, such as the lignans lilifol A and mirandin A, also exhibited cytotoxic effects against MCF-7 cells.^37^ Surprisingly, however, among 56 different extracts, only one was active against MCF-7 cells.

In this context, our study stands out as the first to report a cytotoxic evaluation of *O. villosa*, specifically against MCF-7 breast cancer cells. To date, among the *Ocotea* species investigated, only *O. acutifolia* and isolated compounds from *O. macrophylla* have been assessed against this cell line.^16,37^ Therefore, the results presented here provide new evidence of the antitumor potential of the *Ocotea* genus, suggesting that metabolites present in *O. villosa* may play an important role in the observed cytotoxic activity and encouraging further chemical and biological investigations aimed at identifying the bioactive compounds responsible for this effect.

### 2.2 Clonogenic and Migration Assays

In the control groups, around 600 viable colonies were recorded, while in the groups treated with CE at 100 μg/mL, an average of 315 viable colonies were recorded. Thus, CE decreased colony formation by 50%, as seen in Figure 5A,B.

**Figure 5:**
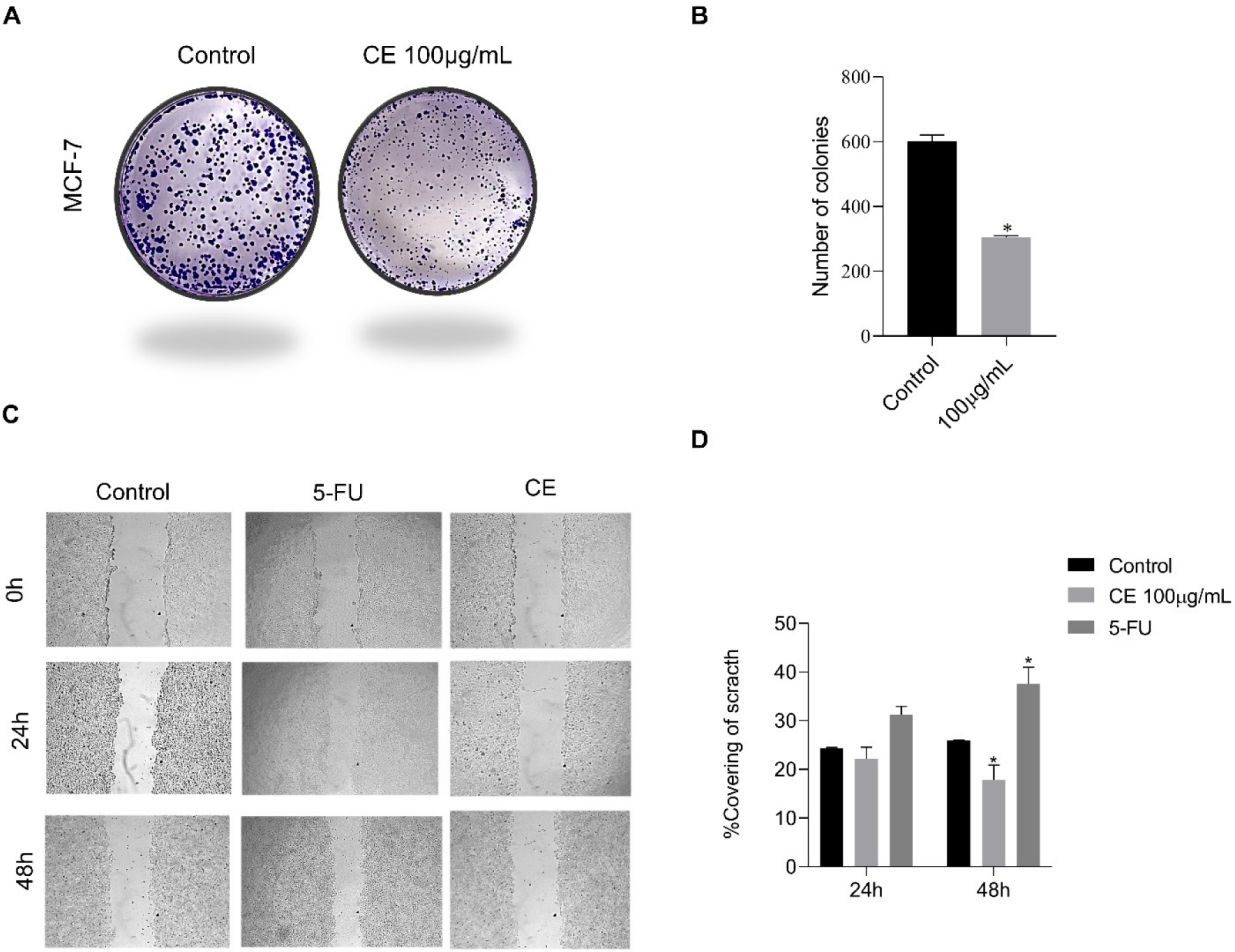
Effect of CE on colony formation and migration of breast cancer cell line MCF-7. (A) Representative images of the clonogenic capacity of MCF-7 cells untreated (control group) and treated with CE 100 μg/mL and (B) quantitative analysis (number of colonies) of MCF-7 cells after 14-day treatment with CE (100 μg/mL). (C) Representative images from the scratch migration assay of MCF-7 cells: untreated (control), treated with CE (100 μg/mL) or with 5-FU (12.5 μg/mL), monitored at 24 and 48 h. (D) Relative migratory capacity of MCF-7 determined by the percentage of scratch area closure using ImageJ software. Results are presented as mean ± SD (*n* = 3). (*) Significant differences from the control group (untreated) were determined according to analysis of variance (ANOVA) followed by Tukey’s posttest, *p < 0.05.

Regarding cell migration (Figure 5C,), there was a significant reduction in the migration of MCF-7 cells treated with CE compared to the control. At the 24-h time point, the percentage of scratch closure was 22% for CE and 24.46% for the control group. At the 48-h time point, the percentage of closure was 17% for CE and 25.91% for the control group. Between 24 and 48 h of CE treatment, there was a 5% reduction in cell migration compared to a 1.45% increase in the control group, suggesting the cells were unable to reestablish intercellular interactions in the CE-treated group during the 48-h treatment period. In comparison, cells treated with 5-FU exhibited 31.22% closure at 24 h and 37.58% at 48 h, approximately 20% higher than CE at the 48-h time point. The results for 5-FU treatment are consistent with previously reported data, in which MCF-7 cells showed approximately 15–16% closure at 24 h and 40% at 48 h. These results indicate a migration inhibitory effect of CE comparable to 5-FU and highlight its potential as a migration-suppressing agent.

Clonogenic assays are important in anticancer drug development as they can evaluate a compound’s ability to inhibit long-term proliferative capacity by revealing its impact on tumor initiation potential, recurrence, and metastasis.^38^ When combined with migration assays, clonogenic assays provide a comprehensive assessment of potential therapeutic efficacy by measuring a compound’s ability to disrupt key cancer hallmarks, such as self-renewal capacity and metastatic spread.^39^ Showing significant inhibition of colony formation and migration, CE is a potential source of antitumor drug candidates.

### 2.3 Apoptosis Assays

The percentage of apoptosis in the control group, CE-treated group, and 5-FU-treated group was 8.83%, 56.59%, and 53.8%, respectively, revealing a significant increase in apoptotic cell death in the treatment groups compared to the control group (Figure 6). CE induced a similar level of apoptosis to 5-FU, which is a positive finding given that 5-FU is an established chemotherapeutic agent.^40^ Thus, CE effects a reduction in MCF-7 cellular proliferation through the mechanism of induction of apoptosis.

**Figure 6:**
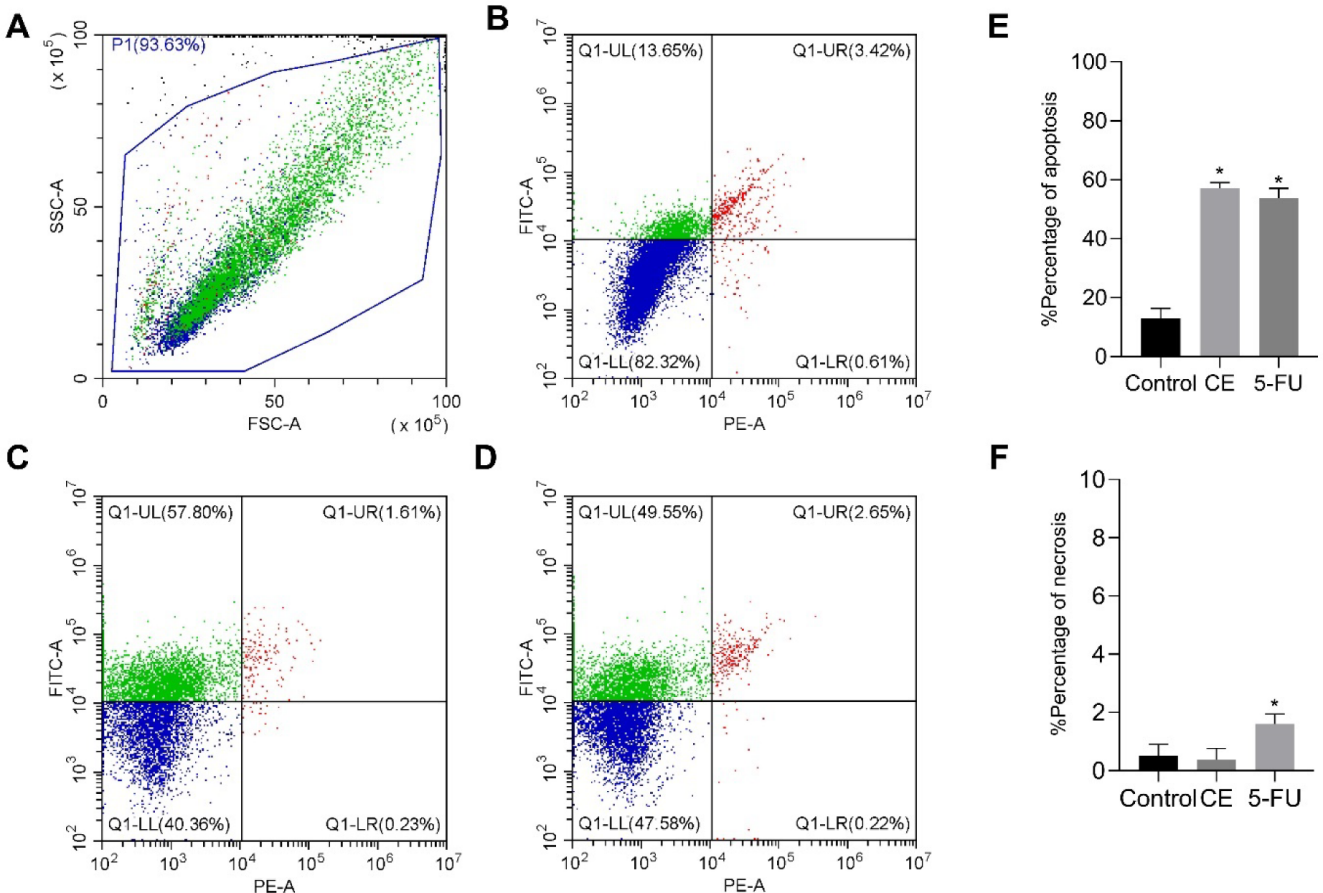
Results of cell death type assays. (A) FSC x SSC diagram of CE. (B) Representative dotplot of one of the triplicates from the control group. (C) Dotplot of one of the triplicates from the group treated with CE. (D) Dotplot of one of the triplicates from the group treated with 5-FU. Graphs of percentage of (E) apoptosis and (F) necrosis. Results are presented as mean ± SD (*n* = 3). (*) Significant differences from the control group (untreated) were determined according to analysis of variance (ANOVA) followed by Tukey’s posttest, *p < 0.05.

The percentage of necrotic cells in the control group, CE-treated group, and 5-FU-treated group was 0.5%, 0.4%, and 1.6%, respectively. Hence, it can be inferred that the predominant type of cell death induced by CE treatment is apoptosis. This is a positive outcome in the investigation of its potential as a drug because cell death by apoptosis is less aggressive than that by necrosis.^41^ Apoptosis is a mechanism of programmed cell death where the cell is induced to die through mechanisms that can be triggered either intrinsically or extrinsically. Ultimately, the apoptotic cell is phagocytosed by macrophages, preventing the release of intracellular molecules into the extracellular environment and thus avoiding the inflammatory process.^41^ When cell death occurs by necrosis, many cells are damaged together. Since it is not self-induced and does not follow a specific sequence of mechanisms, the necrotic process ruptures the cell membrane and releases pro-inflammatory molecules that activate immune system cells, initiating inflammation and characteristic necrotic lesions.^41^ For this reason, the apoptotic process is more efficient for eliminating tumor cells, reducing the likelihood of inflammation and damage to nontumor cells. Inducing apoptosis is a well-established pharmacological strategy in antitumor therapies. Increasing apoptosis-initiating proteins or other molecules is a mechanism being investigated in the development of new drugs that selectively induce apoptosis in cancer cells.^42^

In this context, drugs such as 5-FU stand out for their ability to activate apoptotic pathways in tumor cells, as demonstrated in studies elucidating their mechanisms of action in inducing programmed cell death.^43^ The capacity to trigger apoptosis represents a valuable therapeutic strategy. In 2007, Kline et al. showed that ABT-737, by inhibiting the proteins Bcl-2, Bcl-XL, and Bcl-w, enables tumor cells to undergo apoptosis, thereby reducing tumor growth.^44^ In 2003, Kciuk et al. demonstrated that doxorubicin induces apoptosis through the production of reactive oxygen species (ROS), which damage DNA and mitochondrial membranes, resulting in cytochrome c release and the activation of caspases, including caspase-3 and caspase-8. The induction of apoptosis by antitumor agents is crucial due to its direct relationship with therapeutic efficacy.^45^

### 2.4 Cell Cycle Analysis

The results of cell cycle analysis are displayed in histograms (Figure 7). It can be observed that the control group showed 66.84% of cells in G0/G1, 21.57% in S, and 9.6% in G2/M; the group treated with CE showed 63.08% in G0/G1, 20.94% in S, and 14.41% in G2/M; and the group treated with 5-FU showed 72.58% in G0/G1, 20.32% in S, and 6.43% in G2/M. The cell cycle phase percentages indicate that 5-FU group has a higher percentage of genetic material in the G1 phase compared to the control, suggesting that it influences the cell cycle arrest of MCF-7 cells in the G1 phase. According to Hernández-Vargas et al., 5-FU prevents the progression of the MCF-7 cell cycle in the G1 phase, which supports our findings.^46^ 5-FU is an analog of the molecule uracil with a fluorine atom at position C-5 instead of hydrogen. Its mechanism of action involves inhibiting thymidylate synthase and the incorporation of its metabolites into RNA and DNA.^47^ On the other hand, the results show that CE does not significantly influence the MCF-7 cell cycle, suggesting a mechanism of action different from that observed for 5-FU.

**Figure 7:**
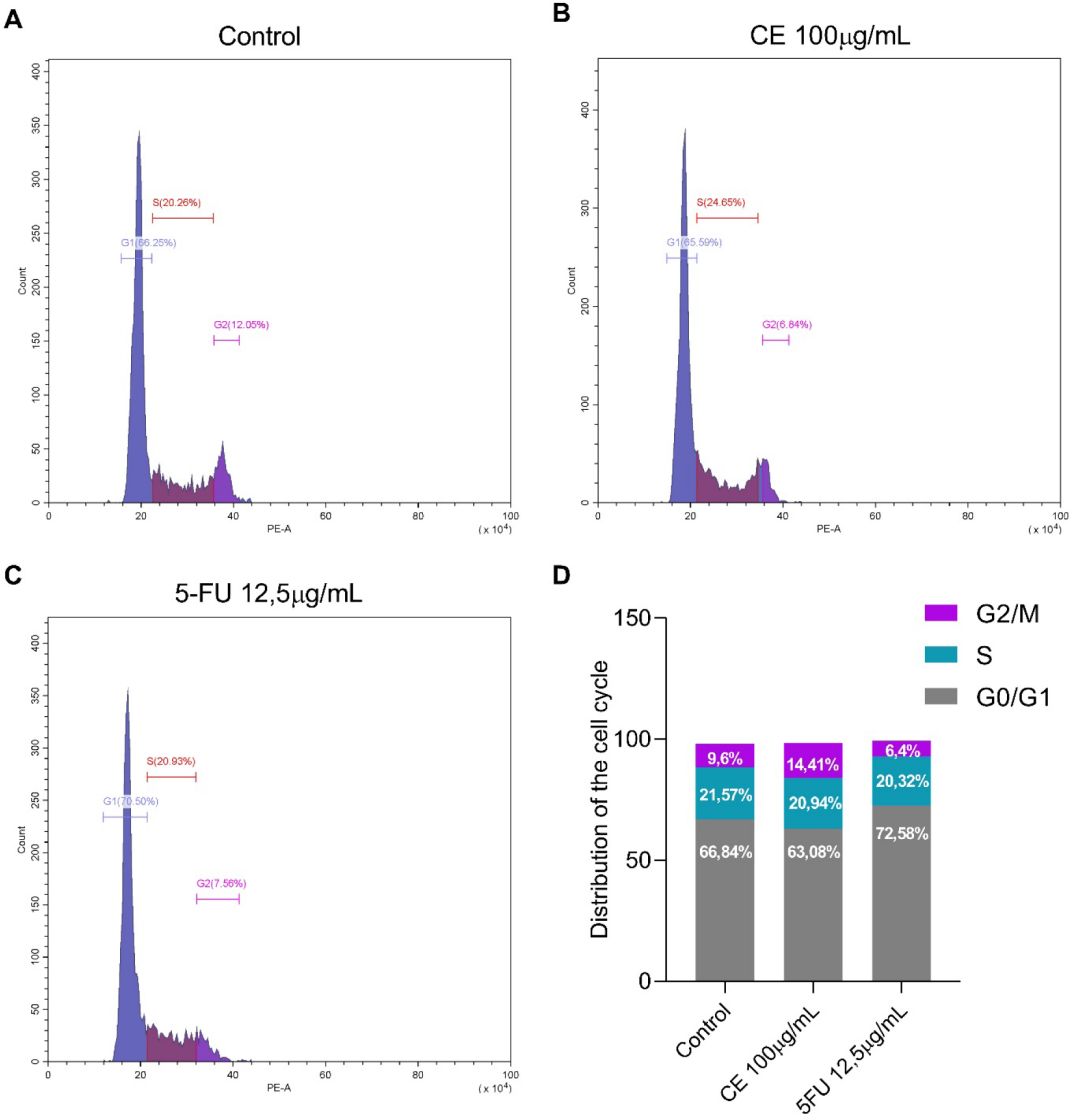
Analysis of the MCF-7 cell cycle. Representative histograms showing cell populations distributed in different phases of the cell cycle: G1 phase (blue), S phase (pink), and G2 phase (purple). (A) Control group, (B) post-treatment with CE at 100 μg/mL, (C) post-treatment with 5-FU at 12.5 μg/mL, for 24 h. (D) Cell cycle analyses. Results are presented as mean ± SD (*n* = 3 biological replicates). (*) Significant differences from the control group (untreated) were determined according to analysis of variance (ANOVA) followed by Tukey’s posttest, *p < 0.05.

### 2.5 RT-PCR

Semiquantitative RT-PCR results show that mRNA levels of caspase-9 (CASP9) were increased after treatment with CE, while CASP8 showed lower expression compared to the control group (Figure 8). Regarding the other markers P16, CCNB1, CDK1, and TP53, no difference was detected between the CE group and the control group (Figure 8).

**Figure 8:**
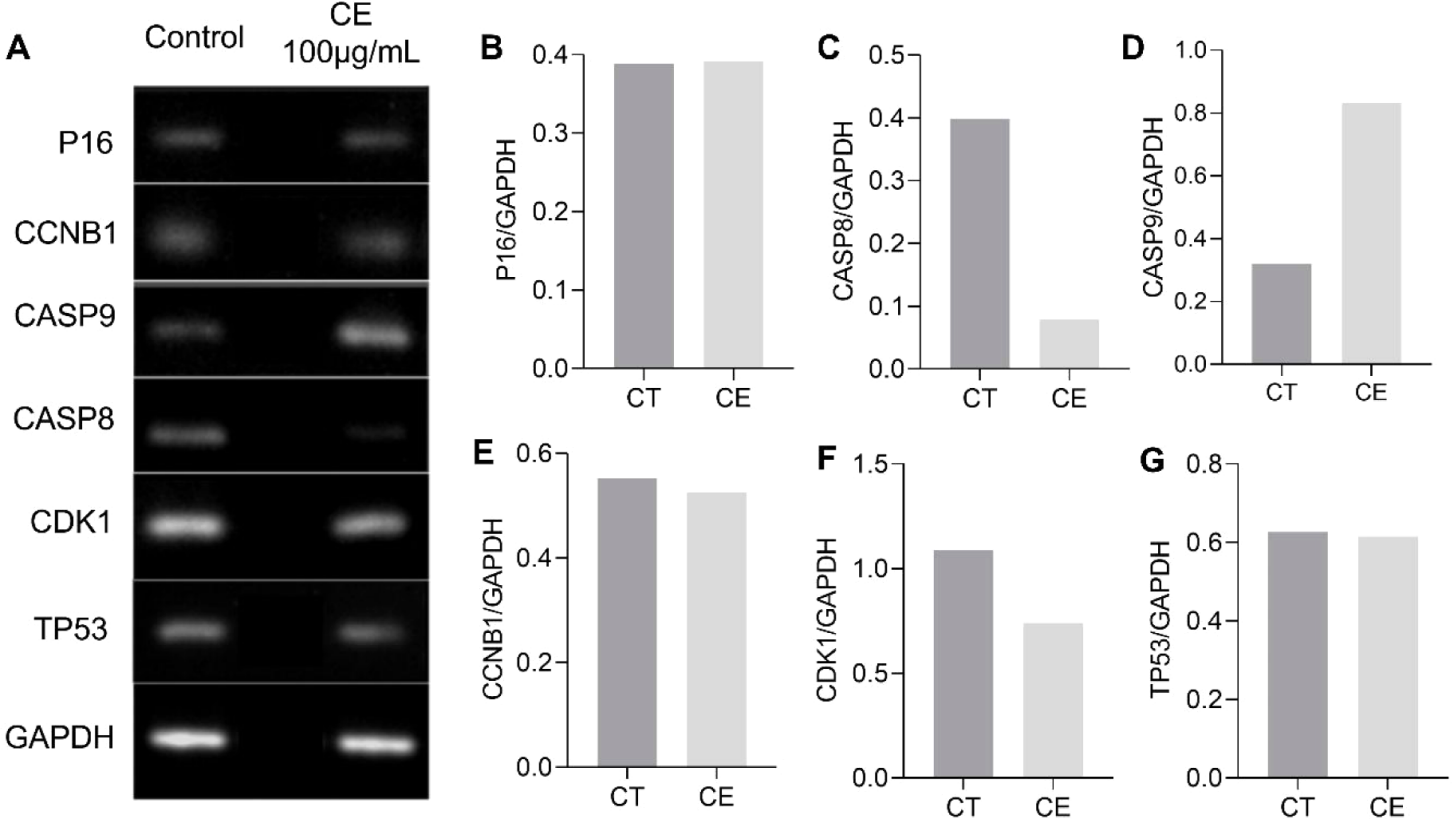
RT-PCR of P16, CCNB1, CASP9, CASP8, CDK1, and TP53 mRNA expression by MCF-7 treated with CE at 100 μg/mL compared to the control group (CT) for 48 h. (A) Representative fragments of agarose gel electrophoresis showing RT-PCR products (amplicons) according to each gene target. (B–G) Graphs representing gene expression by semiquantification of PCR band density of each target gene normalized to glyceraldehyde-3-phosphate dehydrogenase (GAPDH) house-keeping gene, generated through ImageJ software.

Thus, the results suggest that the mechanistic foundation of CE’s antiproliferative activity centers on the activation of the intrinsic apoptotic pathway, as evidenced by the significant upregulation of CASP9 mRNA expression. This finding aligns with extensive literature demonstrating that CASP9 serves as the initiator caspase in the mitochondria-mediated intrinsic apoptosis pathway.^48^ Apoptosis can occur via intrinsic or extrinsic pathways, both of which involve activation by caspases.^48^ The intrinsic pathway is linked to intracellular stress signals, where proteins signal and regulate cell death.^49^ In this pathway, these proteins trigger the release of cytochrome c from the mitochondria, which binds with apoptotic protease-activating factor-1 (Apaf-1) and ATP to form the apoptosome complex, which then recruits and activates procaspase-9 to activate CASP9, which in turn activates the executioner caspases (CASP3, 6, and 7) to execute the final phases of programmed cell death.^49^ The extrinsic pathway is initiated by extracellular ligands binding to membrane receptors that activate CASP8 and CASP9 to trigger the apoptotic cascade. Therefore, caspases are important markers of apoptosis.^49^ The cytotoxic potential of the essential oil of *O. indecora* on cell lines SCC9 (squamous cell carcinoma of the tongue), HT29 (colorectal adenocarcinoma), HepG2 (hepatocarcinoma), and B16F10 (melanoma) was demonstrated through increased CASP3 and CASP7 levels.^50^ In 2011, Chaverri et al. investigated the effects of the essential oils of *O. gomezii* and *O. morae* on tumor cell lines, including MCF-7, and found a reduction in cell viability.^11^

Thus, in contrast to other *Ocotea* species with potential anticancer activity, CE exhibits cytotoxicity in MCF-7 cells, inducing intrinsic apoptosis through activation of CASP9, without detectable changes in cell-cycle distribution. The result is consistent with canonical models in which caspase-9 serves as the initiator that drives executioner-caspase activity and cell death in breast-cancer cells.^51^

### 2.6 Multivariate Statistical Analysis

#### 2.6.1 PCA

PCA is a widely used unsupervised multivariate statistical technique that reduces the dimensionality of complex data sets while preserving variance. It enhances data visualization by revealing clustering patterns, outliers, and overall data structure.^52,53^ Following appropriate data normalization, transformation, and scaling, PCA is applied to assess clustering trends within a data set. In PCA score plots, samples positioned near each other present similar metabolite compositions, whereas those that are farther apart present chemically distinct profiles.^54,55^ The PCA model, computed with eight principal components, yielded *R* ^2^ values of 0.507 (ESI^+^) and 0.521 (ESI^–^), which are considered acceptable for exploratory metabolomics analyses.^52,53,55^

Notably, in the PC1 × PC3, *O. villosa* was clearly separated from other samples falling near the edge or outside of the Hotelling’s T2 ellipse, suggesting distinct metabolic features compared to the other species (Figure 9). The PC3 captures variance that discriminates CE from the background chemical variation across species, whereas PC1 × PC2 is dominated by broad interspecies differences and contributes less to differentiation of CE when compared to the other *Ocotea* species (Figures S1 - S2).

**Figure 9:**
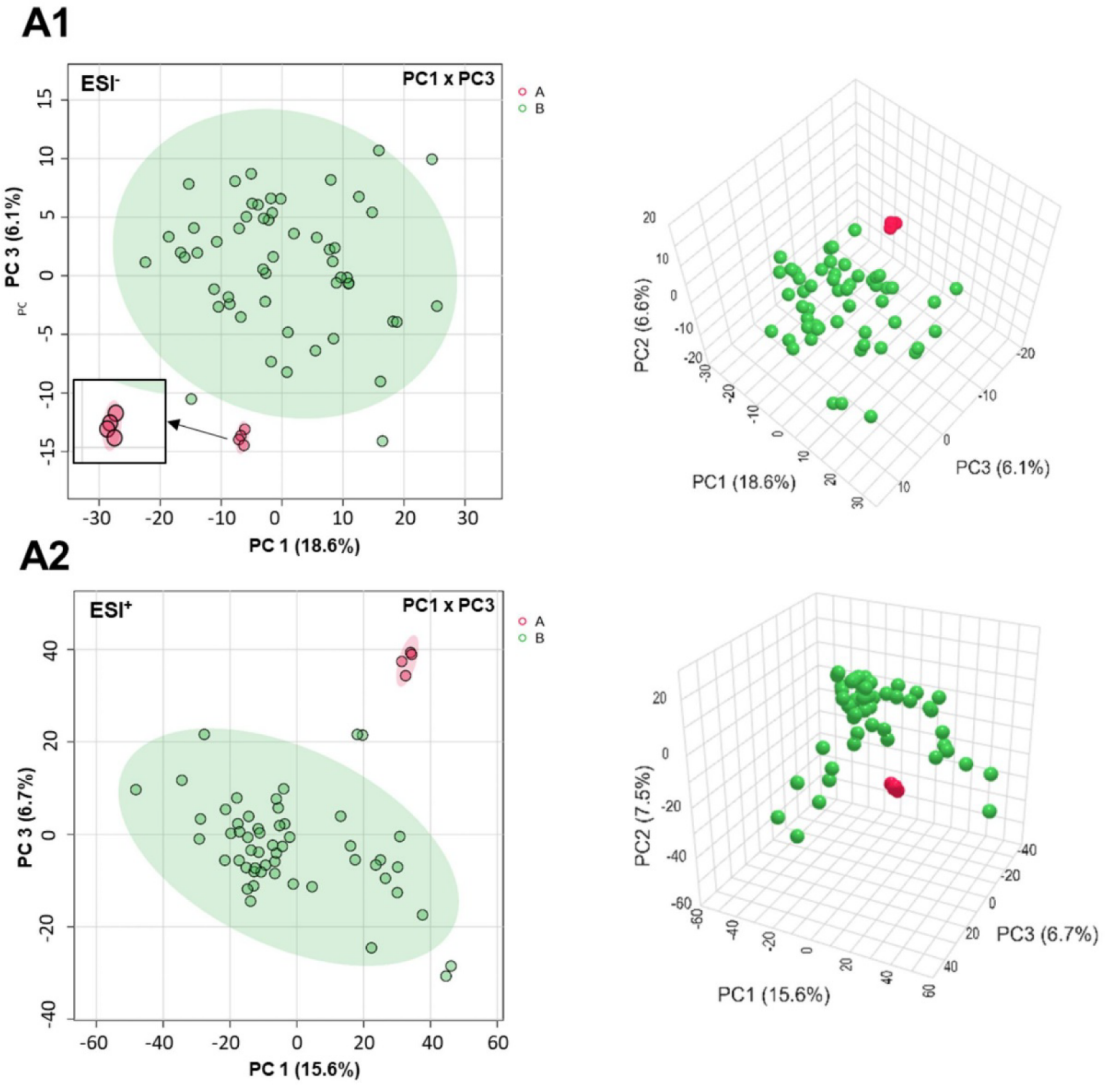
2D score plot (left) of principal component analyses PC1 × PC3 (PCA) and 3D score plot (right) of metabolomic data from UPLC-HRMS analysis in ESI– (A1) and ESI+ (A2) modes. Samples are color-coded as CE (red, 5 analytical replicates) and *Ocotea* spp. (green, single analytical data point per *Ocotea* species). Hotelling’s T2 ellipses in the 2D plots represent the 95% confidence interval for *Ocotea* sp. PCA parameters: 8 components, R^2^ = 0.521 (ESI−) and R^2^ = 0.507 (ESI+). The percentage of variance explained by PC1, PC2, and PC3 is indicated on each axis.

#### 2.6.2 OPLS-DA

After Pareto scaling, the OPLS-DA model for ESI^+^, constructed with three components, demonstrated an excellent fit with *R* ^2^ = 0.978 and *Q* ^2^ = 0.849, yielding an *R* ^2^–*Q* ^2^ difference of roughly 0.13. Similarly, the OPLS-DA model for ESI^–^, also generated with three components, exhibited *R* ^2^ = 0.986, *Q* ^2^ = 0.815, and an *R* ^2^–*Q* ^2^ difference of 0.17. These results indicate a robust classification and high predictive power, as *R* ^2^ represents the proportion of variance explained by the model, while Q^2^ quantifies its predictive ability. Importantly, statistical models with an *R* ^2^–*Q* ^2^ difference exceeding 0.3 are generally considered overfitted,^56^ so the build models are well-fitted and reliable for investigating cytotoxic bioactive markers in the analyzed *Ocotea* samples (Figure 10).

**Figure 10:**
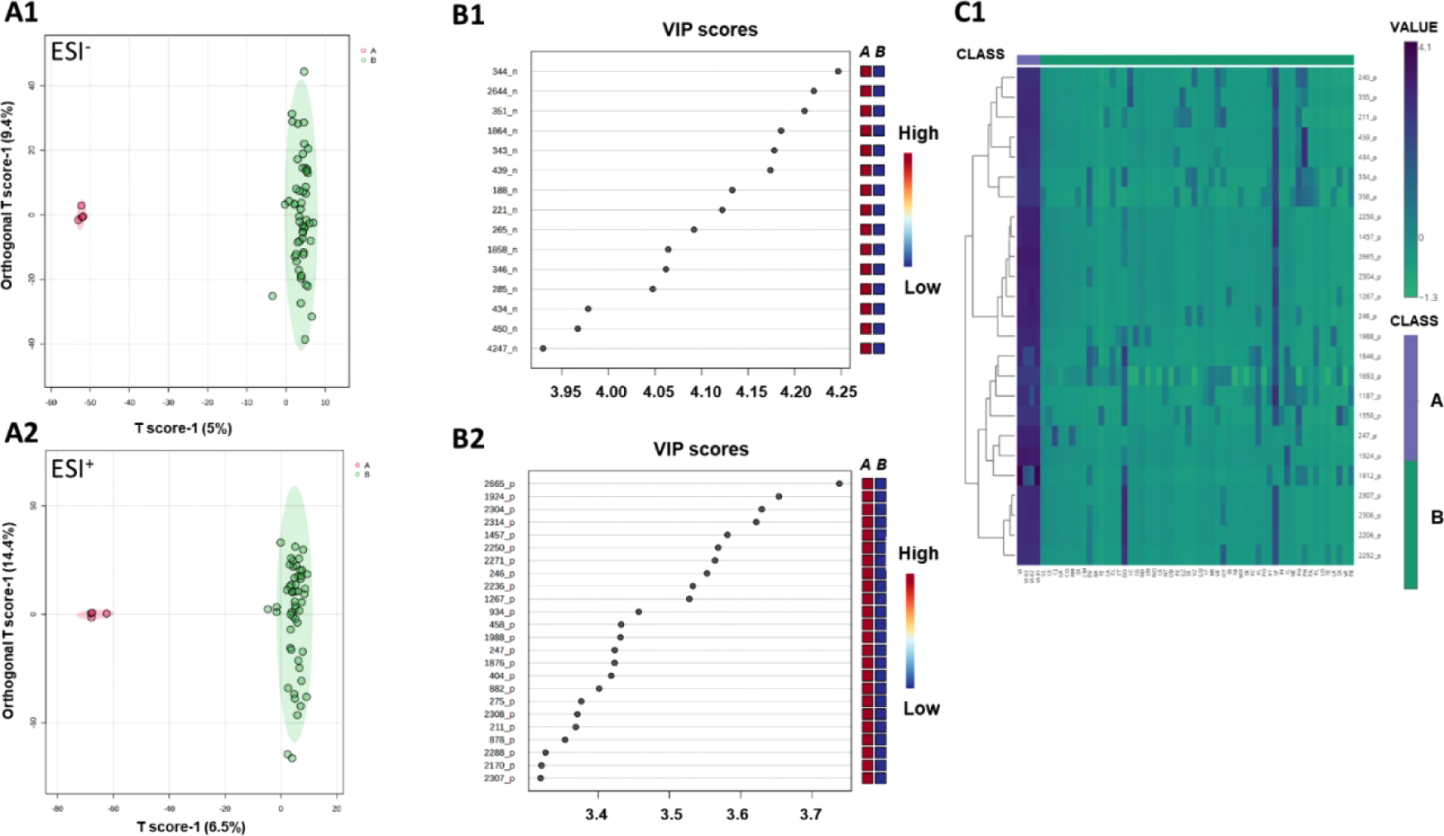
Score plot of OPLS-DA model indicating the discrimination of the active (red, *O. villosa* samples) and inactive sample groups (green, other *Ocotea* spp. samples), sum normalization, log transformation, and Pareto scaling (A1 ESI^–^, A2 ESI^+^). The highest correlation scores are displayed for *m*/*z*/*RT* pairs among the two groups (B1 ESI^–^, B2 ESI^+^). (C1) Heatmap plot for ESI^+^ for 25 differential markers of *O. villosa* (CE) (blue indicates high expression, green indicates low expression) using normalized peak area (square root transformation and Pareto scaled). ESI^+^ OPLS-DA parameters: 3 components, *R* ^2^ = 0.978 and *Q* ^2^ = 0.849; ESI^–^ OPLS-DA: 3 components, *R* ^2^ = 0.986 and *Q* ^2^ = 0.815. The variance explained by PC1 and PC2 is provided in each plot. *O. villosa* (CE), *n* = 5 analytical replicates; other *Ocotea* sp., one LC-MS injection per species.

Additionally, permutation analysis (*n* = 100) indicated that the model’s classification was not due to random chance. Thus, the OPLS-DA was used to identify key metabolic features contributing to cytotoxic activity by analyzing VIP scores (Figure 10). Features (*n* = 27) with VIP scores >3 positively correlated with the *Y* variable were considered highly influential in distinguishing between active and inactive samples, and thus those metabolites likely to be responsible for the observed cytotoxic activity of *O. villosa* samples.^53,56^ These 27 metabolites are statistically valid to be responsible for differences in the cytotoxic activity profile of *O. villosa* and warrant further structural elucidation and bioactivity validation studies (Tables 2 and 3).

**Table 2:** List of Bioactive Markers (VIP > 3) Positively Correlated with the Antiproliferative Activity of *O. villosa* (CE) in the Positive Ionization Mode (ESI+)

ID	RT (min)	Annotation (hits)	Molecular Formula	Observed *m*/*z*	Observed adduct (s)	Error (ppm)
1	1.46	–	C_24_H_31_NO_9_	478.2074	[M + H]^+^	–0.5
2	1.46	–	C_16_H_21_NO_6_	324.1436	[M + H]^+^	–
3	1.63	–	C_46_H_28_N_2_O_7_	721.1975	[M + H]^+^	0.1
4	1.68	laurolitsine	C_18_H_19_NO_4_	627.2700	[2M+H]^+^	0.1
5	1.71	laudanine, 6’-methylreticuline	C_20_H_25_NO_4_	344.1857	[M + H]^+^	–0.1
6	1.74	–	C_18_H_19_NO_4_	627.2709	[2M+H] ^+^	–1.2
7	1.79	–	C_47_H_29_N_3_O_2_	668.2339	[M + H]^+^	0.1
8	1.80	norisoboldine (laurelliptine)	C_18_H_19_NO_4_	314.1376	[M + H] ^+^	3.2
9	2.24	nornuciferine	C_18_H_19_NO_2_	282.1486	[M + H] ^+^	1.0
10	2.30	isomer of 3-hydroxy-6α,7-dehydronuciferine, stephanine	C_19_H_19_NO_3_	310.1443	[M + H] ^+^	–1.7
12	2.48	nuciferine	C_19_H_21_NO_2_	296.1649	[M + H] ^+^	–1.4
13	2.59	isomer of 3-hydroxy-6α,7-dehydronuciferine, stephanine	C_19_H_19_NO_3_	310.1443	[M + H] ^+^	–1.5
14	2.81	3-hydroxy-6α,7-dehydronuciferine	C_19_H_19_NO_3_	310.1445	[M + H] ^+^	–2.1
15	3.01	6α,7-dehydronuciferine	C_19_H_19_NO_2_	294.1491	[M + H] ^+^	–0.6
16	3.12	isothalictrine	C_41_H_44_N_2_O_10_	723.2935	[M − H]^−^	–1.6
18	3.24	guadiscidine	C_19_H_17_NO_2_	292.1324	[M + H] ^+^	2.6
19	3.24	argentinine	C_19_H_21_NO_2_	296.1644	[M + H] ^+^	0.3
20	3.31	–	C_14_H_14_N_2_O_3_	259.1080	[M + H]^+^	–
21	3.70	*N*-butanoyl -(+)-caaverine, guatteriscine	C_21_H_23_NO_3_	338.1747	[M + H] ^+^	1.1
22	3.81	stephenanthrine	C_19_H_19_NO_2_	294.1491	[M + H] ^+^	–0.6
23	3.82	atherosperminine	C_20_H_23_NO_2_	310.1806	[M + H] ^+^	–1.2
24	3.94	stephanine	C_19_H_19_NO3	310.1444	[M + H] ^+^	–2.1
25	3.95	3-methoxynuciferine	C_20_H_23_NO_3_	326.1755	[M + H] ^+^	–1.3
				651.3444	[2M+H] ^+^	–2.2
27	4.69	–	C_19_H_11_NO_6_	350.0662	[M + H] ^+^	–

**Table 3:** List of Bioactive Markers (VIP > 3) Positively Correlated with the Antiproliferative Activity of *O. villosa* (CE) in the Negative Ionization Mode (ESI−)

ID	RT (min)	Annotation (hits)	Molecular Formula	Observed *m*/*z*	Observed adduct (s)	Error (ppm)
1	1.46	-	C_24_H_31_NO_9_	476.1925	[M–H]^−^	0.2
3	1.63	-	C_46_H_28_N_2_O_7_	719.1850	[M–H]^−^	–
4	1.68	laurolitsine	C_18_H_19_NO_4_	938.3889 625.2553	[3M–H]^−^ [2M–H]^−^	–2.2 0.4
7	1.79	-	C_47_H_29_N_3_O_2_	666.2211	[M–H]^−^	–
8	1.80	norisoboldine (laurelliptine)	C_18_H_19_NO_4_	625.2553 938.3889	[2M-H] ^–^ [3M-H] ^–^	0.0 0.0
11	2.32	-	C_32_H_16_N_2_O_2_	459.1125	[M–H]^−^	–
16	3.12	isothalictrine	C_41_H_44_N_2_O_10_	723.2935	[M–H]^−^	–1.6
17	3.12	ocoteine *N*-oxide	C_21_H_23_NO_6_	384.1453	[M–H]^−^	–0.2
26	4.45	-	C_20_H_26_O_8_	393.1541	[M–H]^−^	–

Furthermore, a fold-change univariate statistical analysis was incorporated into the analyses of both positive and negative ionization modes data, confirming a consistent pattern of differential metabolic expression of *O. villosa* compared to other *Ocotea* species. A subset of 27 metabolites was significantly upregulated in *O. villosa*, as indicated by Log2 fold change (Log2FC > 5, colored in red). Similarly, several metabolites were strongly downregulated in *O. villosa* (Log2FC < −5, colored in blue), indicating their higher abundance in other *Ocotea* species (Figure 11A,B).

**Figure 11:**
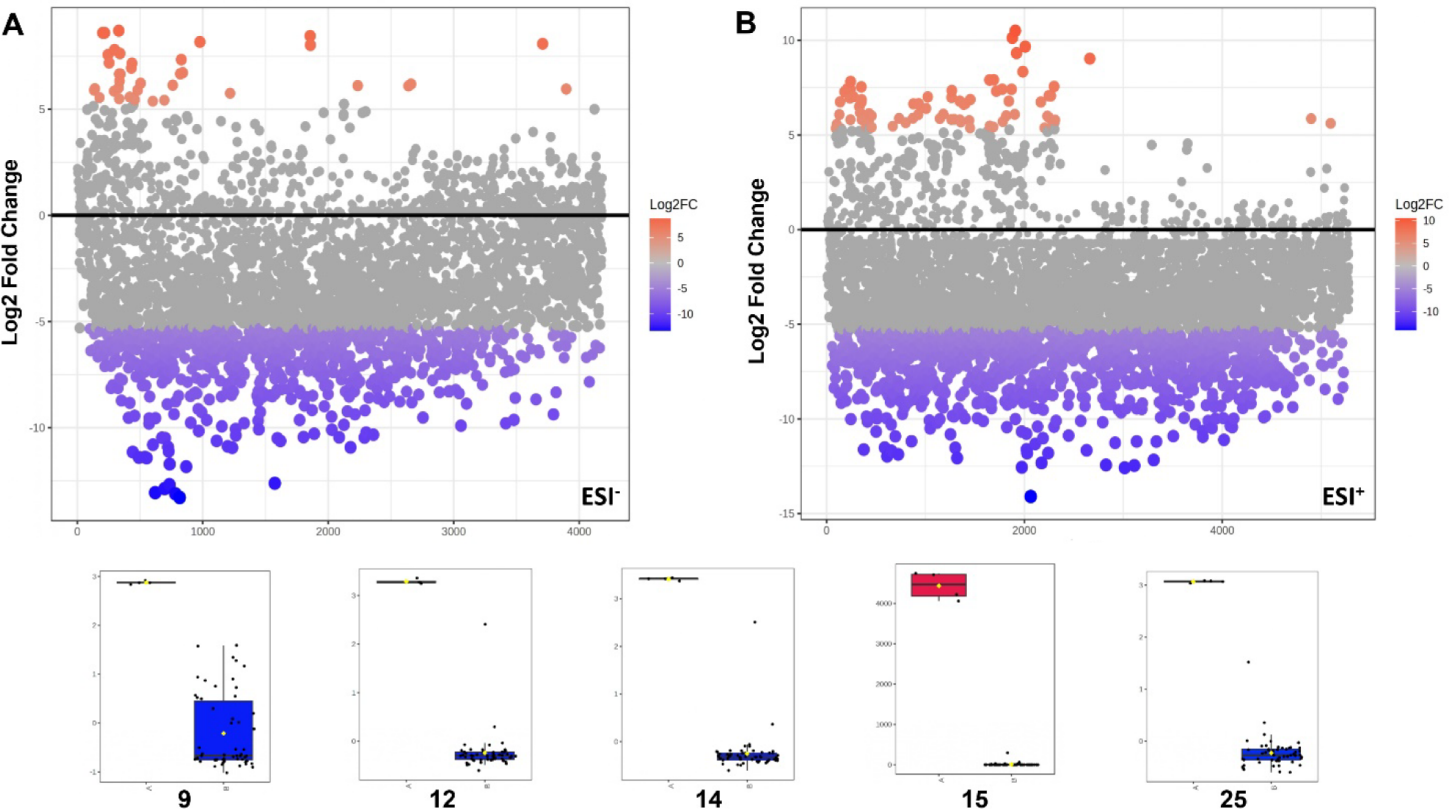
Fold-change plots were generated for (A) negative and (B) positive ionization mode using the original OPLS-DA data set obtained from UPLC-HRMS data processing. The statistical cutoff for considering metabolites as significantly different was defined as fold-change >5. Examples of bar graphs from the main variable importance in projection scores (VIPs).

### 2.7 Bioactive Markers Annotation

The chromatographic profile of *O. villosa* exhibited notable differences compared to the other *Ocotea* species analyzed, particularly in the 1–4 min retention time range, where intense peaks were observed and mainly chemically annotated as alkaloids (Figure 12).

**Figure 12:**
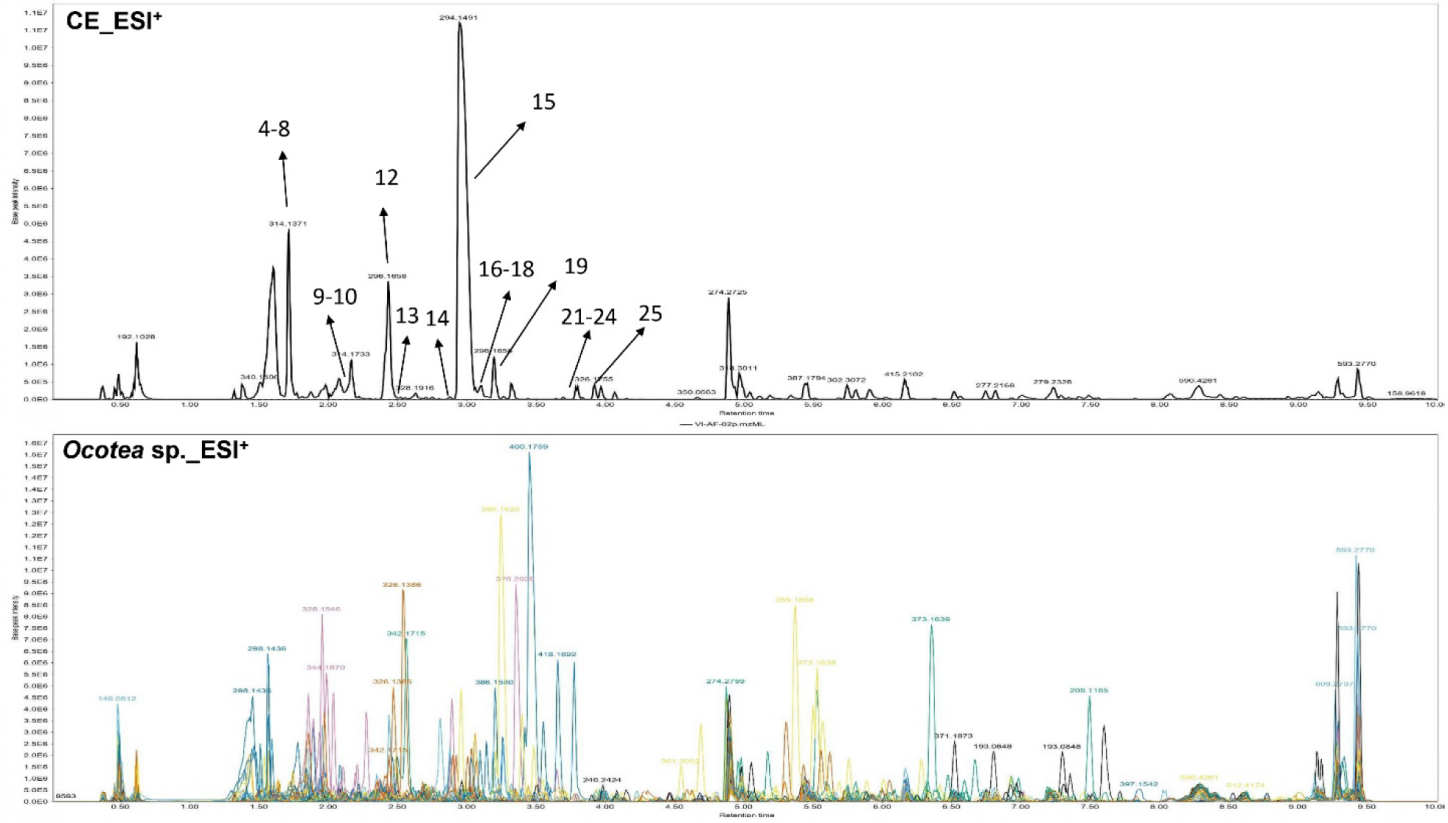
LC-HRMS metabolic fingerprints of base peak intensity (BPI) chromatograms in the positive mode showing the overlapped metabolic fingerprint of *O. villosa* and other *Ocotea* species.

Key features identified through variable importance in projection (VIP) statistical analysis scores were further investigated and putatively annotated based on monoisotopic mass screening against chemical databases (Tables 2 and 3). Most of the annotated natural products belong to the alkaloid class (*n* = 19), particularly from the isoquinoline family, such as aporphinoids (including aporphines and noraporphines), phenantrenes, and benzylisoquinolines.

Aporphinoid alkaloids have been associated with promising bioactivities, particularly in oxidative stress-related diseases, including various cancers.^57^ These compounds are known to exhibit prominent anticancer activity, with 1,2-methylenedioxy and *N*-methylated groups being key molecular features associated with their antiproliferative properties. Several of the annotated alkaloids in *O. villosa* exhibited these molecular characteristics, further supporting their potential as bioactive markers.^58,59^

The molecular mechanisms of aporphinoid alkaloids are primarily associated with the induction of cancer cell apoptosis, inhibition of cell proliferation, DNA topoisomerase inhibition, and suppression of epidermal growth factor receptor (EGFR) tyrosine kinase activity.^58^ Therefore, our findings align with the mechanisms of action of the compounds present in CE. It is noteworthy that nuciferine (ID 12) and its derivatives, such as nornuciferine (ID 9), 3-hydroxy-6α,7-dehydronuciferine (ID 14), 6α,7-dehydronuciferine (ID 15), and 3-methoxynuciferine (ID 25), were among the features with the highest correlated VIP scores (Figure 13). These metabolites were chemically annotated using MS^1^ and MS^2^ data obtained via MS^E^ data-independent acquisition. For 5 and 7 annotation confidence reached Metabolomic Standard Initiative (MSI) Level 2, as fragmentation spectra were directly compared with spectral libraries (GNPS). Besides, according to current literature,^60^ and based on the same fragmentation pattern observed and diagnostic ions (e.g., *m*/*z* 165) the derivatives 9, 10, and 18 were also proposed (Table 4). This finding is particularly relevant given the well-documented anticancer properties of nuciferine,^61,62^ including its reported cytotoxic effects against breast cancer cell lines.^63,64^

**Figure 13:**
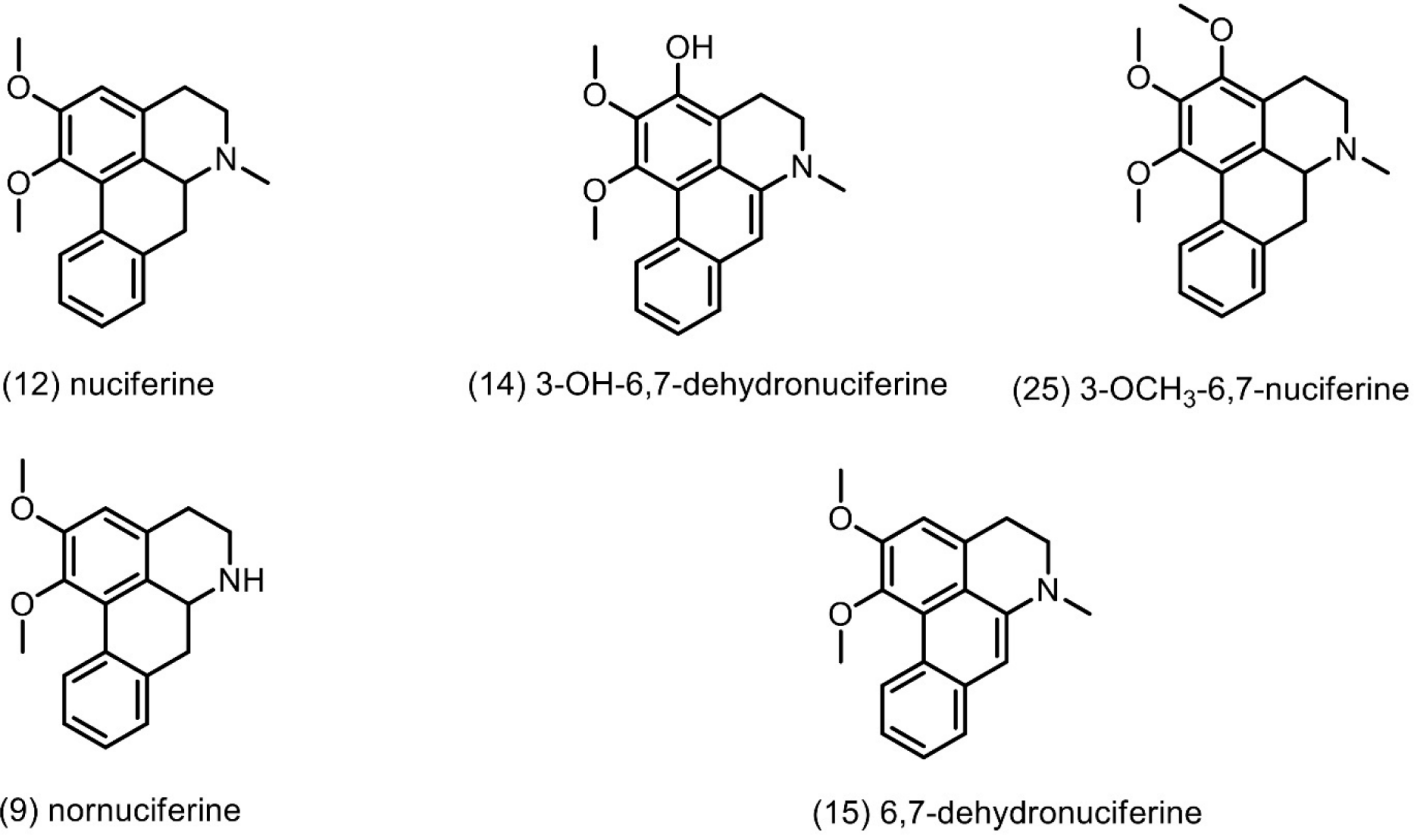
Nuciferine and derivatives chemically annotated, at Metabolomic Standard Initiative (MSI) confidence Level 2, with the highest correlated VIP scores.

**Table 4:** List of Nuciferin Derivatives (VIP > 3) Annotated with Level 2 of Confidence According to the Metabolomic Standard Initiative (MSI) from *O. villosa* (CE) in Positive Ionization Mode (ESI^+^), Correlated with Antiproliferative Activity^a^

ID	Observed *m*/*z*	Adduct	MS/MS fragments	Reference	InChIKey
9	282.14870	[M + H]^+^	152.06110; 165.06844; 178.07695; 179.08540; 189.07059; 191.08656; 202.07614; 207.08092; 235.07558; 265.12411	GNPS	QQKAHDMMPBQDAC-AWEZNQCLSA-N
12	296.16469	[M + H]^+^	165.06844; 178.07695; 179.08540; 189.07059; 190.07584; 191.08656; 207.08864; 219.08057; 265.12079	GNPS	ORJVQPIHKOARKV-OAHLLOKOSA-N
14	310.1443	[M + H]^+^	165.06844; 178.07695; 191.08656; 207.08864; 235.07558; 263.12079;	Proposed^b^	MBOVAZNWFZXHMQ-UHFFFAOYSA-N
15	294.14903	[M + H]^+^	165.06844; 193.09901; 207.07799; 218.07071; 219.07755; 235.07558; 249.09068; 263.07019	Proposed^b^	JBGSWIBJAGBGOP-UHFFFAOYSA-N
25	326.1755	[M + H]^+^	165.06844; 178.07695; 189.07059; 191.08656; 207.08864; 235.07558; 265.12079	Proposed^b^	NQMHAYITAGKJMF-UHFFFAOYSA-N

a Note: GNPS (Global Natural Product Social Molecular Networking) spectral library were used for data comparison.

b Fragment ions were proposed based on chemical knowledge of MS fragmentation and observed MS^E^ spectra.

Nuciferine is a well-known bioactive alkaloid found in *Nelumbo nucifera* (lotus), a key plant in Asian traditional medicine,^65^ and has not been previously isolated in *Ocotea* species, though it was recently annotated in *O. diospiriofolia*.^66^ However, the genus *Ocotea* is characterized by a highly specialized metabolism within the isoquinoline alkaloid biosynthetic pathway, with aporphinoids being the predominant class of alkaloids identified in this group.^66^ In addition, some derivatives, including 6α,7-dehydronuciferine, nornuciferine, and 3-methoxynuciferine, have already been isolated from *Ocotea* species,^17^ which supports the annotation of these compounds in CE.

Thus, in this work, nuciferine and its derivatives were annotated in CE under the MSI Level 2 criteria, which were characterized by MS^1^ and MS^2^ data match.^67,68^ Besides, as these metabolites are known to be produced by plants from the *Ocotea* genus, it enhances the confidence of the proposed metabolite annotation. To the best of our knowledge, this represents the third report of the alkaloid nuciferine in the genus *Ocotea*. Prior *Ocotea* isolation reports document nuciferine derivatives only (e.g., nornuciferine, 6α,7-dehydronuciferine, 3-hydroxy-6α,7-dehydronuciferine, and 3-methoxynuciferine).^17,69^ Unequivocal confirmation in future studies, through MSI Level 1 annotation, requires coanalysis with an authentic standard (matching RT and MS/MS under identical conditions in the same analysis) or by isolation and full characterization (orthogonal spectroscopic data). Further studies on these candidate biomarkers could provide novel insights supporting future investigations into novel natural antiproliferative agents. Furthermore, metabolomic profiling revealed alkaloid enrichment with aporphine/nuciferine-type annotations, providing a chemical context for the observed intrinsic apoptosis, in line with reports that natural products can reduce breast cancer cell survival via apoptotic mechanisms.^70,71^

## 3 Conclusion

This study screened crude extracts of 56 *Ocotea* species for in vitro cytotoxic activity against MCF-7 breast cancer cells. Among the tested extracts, only that of CE exhibited significant cytotoxicity against MCF-7 (IC_50_ = 100.00 ± 6.06 μg/mL). Metabolomic profiling revealed high levels of alkaloids in CE, including major aporphine compounds such as nuciferine-derived alkaloids. Its high content of bioactive aporphine alkaloids suggests that it could serve as a natural source of promising compounds and chemical scaffolds for the future development of breast cancer therapies. Mechanistic investigation demonstrated that CE induces selective cytotoxicity, inhibits cell colony formation by 50%, and reduces cell migration by 5%. Moreover, CE induced apoptosis in approximately 56.6% of an MCF-7 cell population, without significantly altering the cell cycle. Notably, CASP9 mRNA upregulation suggests that CE acts through activation of the intrinsic apoptosis pathway. These findings are the first to demonstrate the antiproliferative effects of *O. villosa*, highlighting it as a promising plant matrix for bioprospecting in the field of natural product-based anticancer drug discovery. However, this study relied exclusively on in vitro models, and the specific mechanisms of action of individual compounds were not fully elucidated. Therefore, further studies should be performed to investigate in detail the molecular mechanisms underlying the antiproliferative antitumor effects of CE and its isolated active metabolites, including at the protein level and through other techniques, and to evaluate their efficacy and safety in in vivo models. Addressing these aspects will be essential to advance the potential therapeutic applications of these compounds. Furthermore, investigating the combination of the extract or its active constituents with reference chemotherapeutic drugs for combined breast cancer therapy, and studying the bioactivity of CE and its metabolites in other breast cancer cell lines, are important perspectives of this work.

## 4 Materials and Methods

### 4.1 Plant Material

The access to plant samples (leaves) of the 56 different *Ocotea* spp. used in this study (Table 5) was registered in the National System for Governance of Genetic Heritage and Associated Traditional Knowledge (SisGen # A5A8F67). All species had vouchers deposited in the herbarium at UNIFAL-MG, and only the quantity of leaves necessary for the study was collected, which did not compromise their respective survivals. The samples were dried at 40 °C. Some samples were donated from the OUPR herbarium (Federal University of Ouro Preto—UFOP) and CESJ herbarium (Federal University of Juiz de Fora—UFJF), also in the minimum required quantity, without any prejudice to the deposited material.

**Table 5:** Species of the *Ocotea* Genus Evaluated in the MCF-7 Cell Viability Screening

1	*Ocotea amazonica* (Meiss) Mez	29	*Ocotea nitida* (Meisn.) Rohwer
2	*Ocotea brachybotrya* (Meisn.) Mez	30	*Ocotea nitidula* (Nees et Mart. ex Ness)
3	*Ocotea bicolor* Vattimo-Gil	31	*Ocotea notata* (Nees & Mart.) Mez
4	*Ocotea caesia* Mez	32	*Ocotea nutans* (Nees) Mez
5	*Ocotea catharinensis* Mez	33	*Ocotea odorifera* Vell. Rohwer
6	*Ocotea cujumary* Mart.	34	*Ocotea paranaensis* Brotto, Baitello, Cervi & E.P.Santos
7	*Ocotea cernua* (Nees) Mez	35	*Ocotea percoriacea* Kosterm.
8	*Ocotea corymbosa* (Meisn.)Mez	36	*Ocotea pomaderroides* (Meisn.) Mez
9	*Ocotea complicata* (Meisn.) Mez	37	*Ocotea porosa* (Nees & Mart.) Barroso
10	*Ocotea dispersa* (Nees & Mart.) Mez	38	*Ocotea pretiosa* (Nees) Mez
11	*Ocotea diospyrifolia* (Meisn.) Mez	39	*Ocotea puberula* (Rich.) Nees
12	*Ocotea divaricata* (Nees) Mez	40	*Ocotea pulchea* Vattimo-Gil
13	*Ocotea elegans* Mez	41	*Ocotea pulchella* (Nees & Mart.) Mez
14	*Ocotea felix*Coe-Teix.	42	*Ocotea minarum* (Nees & Mart.) Mez
15	*Ocotea glauca* (Nees & Mart.) Mez	43	*Ocotea nummularia*
16	*Ocotea glaucina* (Meisn.) Mez	44	*Ocotea nectandrifolia* Mez
17	*Ocotea glaziovii* Mez	45	*Ocotea pulchra* Vattimo-Gil
18	*Ocotea guianensis* Aubl.	46	*Ocotea spectabilis* (Meisn.) Mez
19	*Ocotea hypoglauca* (Nees & Mart.) Mez	47	*Ocotea spixiana* (Nees) Mez
20	*Ocotea indecora* (Schott) Mez	48	*Ocotea tabacifolia* (Meisn.) Rohwer
21	*Ocotea kuhlmannii*Vattimo-Gi	49	*Ocotea tenuiflora* (Nees) Mez
22	*Ocotea lanata* (Nees & Mart.) Mez	50	*Ocotea teleiandra* (Meisn.) Mez
23	*Ocotea lanceolata* (Nees) Nees	51	*Ocotea tristis* (Nees & Mart.) Mez
24	*Ocotea lancifolia* (Schott) Mez	52	*Ocotea vaccinioides* (Meisn.) Mez
25	*Ocotea langsdorffii* (Meisn.) Mez	53	*Ocotea variabilis* Mart.
26	*Ocotea laxa* (Nees) Mez	54	*Ocotea velloziana* (Meisn.) Mez
27	*Ocotea lobbii* (Meisn.) Rohwer	55	*Ocotea velutina* (Nees) Rohwer
28	*Ocotea longifolia* Kunth	56	*Ocotea villosa* Kosterm.

### 4.2 Crude Extract Preparation

The dried samples were cryogenically ground in a mortar with a pestle and liquid nitrogen until pulverized. Subsequently, powdered material (20 mg per sample) was extracted with ethanol/water (1.7 mL, 7:3 v/v). The suspensions were sonicated in a heated ultrasonic bath at 35 °C for 15 min (170 W, 50 kHz, L100 Schuster), followed by centrifugation at 22 °C and 12,000 rcf. The resulting supernatants were subsequently partitioned with hexane to remove nonpolar substances. The 56 extracts were filtered through a hydrophobic polytetrafluoroethylene (PTFE) filter (pore size 0.22 μm) and dried using a vacuum centrifuge for 3 h at 40 °C. The 56 dried extracts were stored at −20 °C until further analysis (Figure 1), screening of activity against the breast cancer cell line (Section 4.4), and metabolomics (Section 4.11). The extracts were solubilized in water for metabolomics analysis and 1% DMSO for biological assays, both at 1 mg/mL.

### 4.3 Cell Lines and Culture Media

Breast carcinoma (MCF-7) and human skin fibroblast (CCD-1059Sk) cell lines were used in the study. CCD-1059Sk cells were maintained in Dulbecco’s Modified Eagle’s Minimum Essential Medium (DMEM, Vitrocell-Embriolife, São Paulo, Brazil) supplemented with 10% fetal bovine serum (FBS, Cultilab, São Paulo, Brazil). MCF-7 cells were cultivated in DMEM supplemented with 10% FBS and 0.01 μg/mL human insulin (Novolin N, NPH, Novo Nordisk Farm. do Brasil Ltd.a, São Paulo, Brazil). Cells were grown in a humidified atmosphere of 95% air and 5% CO_2_ at 37 °C.

### 4.4 Screening of MCF-7 Cell Viability

MCF-7 cells were plated in a 96-well plate at a density 5 × 10^3^ cells per well and maintained at 37 °C in a humidified atmosphere with 5% CO_2_. The MCF-7 cells were exposed to 56 *Ocotea* spp. extracts at a concentration of 40 μg/mL in DMEM medium for 48 h (Figure 1). After 48 h, cell viability was assessed using the MTT (3-(4,5-dimethylthiazol-2-yl)-2,5-diphenyltetrazolium bromide, Sigma-Aldrich) assay following the manufacturer’s instructions. The treatment medium was removed, and MTT solution (5 mg/mL) was added to each well. The plate was then incubated for 4 h at 37 °C in a 5% CO_2_ atmosphere. The resulting formazan crystals were dissolved in isopropanol containing 0.1 N HCl. Absorbance was measured at 570 nm with background correction at 645 nm using a Multiskan GO (Thermo Scientific). Cell viability was calculated as a percentage relative to the untreated control cells.

### 4.5 Mechanism of Action Investigation

#### 4.5.1 MCF-7 Cell Viability—*Ocotea villosa* Active Sample

The sample that showed the most significant cytotoxic activity in the screening phase (Section 2.1, Figure 2, *p* < 0.05 compared to nontreated control group) was selected for investigation of its mechanism of action.

Thus, MCF-7 and CCD-1059Sk cells were treated with increasing concentrations of CE, the most promising extract identified in the screening phase (CE 12.5–200 μg/mL for MCF-7 and 25–400 μg/mL for CCD-1059Sk). 5-Fluorouracil (5-FU) was used as the positive control and evaluated across increasing concentrations (3.125–50 μg/mL). After 48 h of treatment, cell viability was assessed using the MTT assay described in Section 4.4. The IC_50_ values were determined through nonlinear regression analysis using GraphPad Prism 8. All MTT assays were performed in three independent biological replicates (*n* = 3).

#### 4.5.2 Trypan Blue Exclusion Test

MCF-7 cells were plated in a 24-well plate at a concentration of 5 × 10^3^ cells per well. The cells were exposed to CE at concentrations of 150, 100, and 50 μg/mL for 24, 48, and 72 h. Following treatment, the cells were detached using trypsin. A 0.4% trypan blue solution was mixed with the cell suspension in DMEM at a 1:1 ratio. The cell mixture was then analyzed using a hemocytometer under a light microscope to count the cells. Both viable cells (which remained unstained) and nonviable cells (which absorbed the blue stain) were counted and recorded. This assay was performed in three independent biological replicates (n = 3).

#### 4.5.3 Clonogenic Assay

MCF-7 cells were plated in a 6-well plate at a density of 1000 cells per well. The cells were treated with CE at a concentration of 100 μg/mL for 48 h and then cultured in an extract-free medium at 37 °C in a humidified atmosphere containing 5% CO_2_ for an additional 14 days. After this period, the colonies were fixed with 10% paraformaldehyde and subsequently stained with crystal violet. Colonies containing more than 50 cells were identified and counted through direct visual observation using an optical microscope.^72^ This assay was performed in three independent biological replicates (*n* = 3).

#### 4.5.4 Scratch Assay

To evaluate cell migration ability, MCF-7 cells were seeded in a 6-well plate at a density of 5 × 10^5^ cells per well and incubated for 24 h at 37 °C in a humidified 5% CO_2_ environment to achieve a fully confluent monolayer. A straight scratch was then made in the cell monolayer of each well using a sterile tool. The wells were gently rinsed twice with PBS to remove detached cells. Following this, the cells were treated with CE at a concentration of 100 μg/mL and 5-FU at 12.5 μg/mL, the latter serving as a positive control. Images of the scratch in each well were captured at 0, 24, and 48 h post-treatment using an inverted optical microscope (Physis) equipped with an anatomical camera (Opton 5150). The scratch area at each time point was measured using ImageJ software, and the percentage of scratch closure was calculated using the formula: scratch closure (%) = [(scratch area at 0 h – scratch area at treatment time) ÷ scratch area at 0 h] × 100. This assay was performed in three independent biological replicates (*n* = 3).

#### 4.5.5 Apoptosis Detection

To determine the type of cell death in MCF-7 breast cancer cells, the Annexin V and propidium iodide (PI) staining method was employed using the Alexa Fluor 488 Annexin V/PI kit (Invitrogen) following the manufacturer’s protocol. Flow cytometry analysis was conducted using a CytExpert for DxFlex system (Beckman Coulter). The cells were seeded at a density of 5 × 10^5^ cells per well in a 24-well plate and incubated at 37 °C in a 5% CO_2_ atmosphere for 24 h. Following incubation, the cells were treated with 100 μg/mL of CE and 12.5 μg/mL of 5-FU. After 24 h, the culture medium was collected, and the cells were washed, detached using trypsin, centrifuged, and resuspended for cell counting. A minimum of 1.5 × 10^5^ cells/mL were then incubated with a staining buffer. After a 20 min incubation period at room temperature, the samples were analyzed by flow cytometry. Ten thousand events were recorded, and the results were presented graphically as the percentage of apoptotic and necrotic cells. This assay was performed in three independent biological replicates (n = 3).

#### 4.5.6 Cell Cycle Analysis

MCF-7 breast cancer cells were seeded at a concentration of 4 × 10^5^ cells per well in 24-well plates (Sarstedt) and incubated for 24 h at 37 °C in a 5% CO_2_ environment. After incubation, the medium was replaced with a treatment medium containing 100 μg/mL of CE and 12.5 μg/mL of 5-FU, and the cells were exposed to this medium for an additional 24 h. Following treatment, the culture medium was collected and transferred to a microtube. The cells were washed with PBS, centrifuged, and the supernatant was discarded. The resulting cell pellet was resuspended in 25 μL of cold 1× PBS and 230 μL of absolute ethanol. The tubes were then incubated at 4 °C for 20 min and centrifuged at 111 rcf for 5 min. The pellet was subsequently resuspended in a cell cycle buffer solution (containing 30 μg/mL of PI and 100 μg/mL of RNase free of DNase in PBS) and incubated for 45 min at room temperature in the dark. Finally, the samples were analyzed using flow cytometry (CytExpert for DxFlex, Beckman Coulter), with a minimum of 10,000 events recorded. This assay was performed in three independent biological replicates (*n* = 3).

#### 4.5.7 RT-PCR

mRNA levels of tumor protein p53 (TP53), caspase 9 (CASP9), caspase 8 (CASP8), cyclin dependent kinase 1 (CDK1), cyclin B1 (CCNB1) and cyclin dependent kinase inhibitor 2A (CDKN2A) were analyzed by conventional PCR. The gene expression was assessed in MCF-7 cells using glyceraldehyde-3-phosphate dehydrogenase (GAPDH) as a reference gene.

MCF-7 cells were seeded at a concentration of 1 × 10^5^ cells per well in 6-well plates (Sarstedt) and incubated for 24 h at 37 °C in a 5% CO_2_ environment. After incubation, the medium was replaced with a treatment medium containing CE at a concentration of 100 μg/mL for 48 h, alongside an untreated control group. This assay was performed in three biological replicates (*n* = 3). Following the treatment period, total RNA was isolated using TRIzol reagent in accordance with the manufacturer’s protocol (Invitrogen). The RNA samples were then treated with DNase (RNase-free, Promega, USA) to remove any genomic DNA contamination. The concentration of total RNA (ng/mL) was quantified using a NanoDrop ND-1000 microspectrophotometer. Subsequently, cDNA synthesis was carried out using the RevertAid H Minus First Strand cDNA Synthesis Kit (Thermo Scientific), following the provided instructions.

Next, the PCR reaction was performed using the GoTaq DNA polymerase (Promega) using a thermocycler (Veriti 96, Applied Biosystems) following the manufacturer’s protocol and instructions. PCR amplification was performed for the gene targets using the specific primers TP53 (NM_000546.6; F:TGCTCAGATAGCGATGGTCT; R:TAGGGCACCACCACACTATG), CASP9 (NM_001229; R: GTTTGAGGACCTTCGACCAGCT; F:CAACGTACCAGGAGCCACTCTT), CASP8 (NM_001080125.2; F:AGAAGAGGGTCATCCTGGGAGA; R: TCAGGACTTCCTTCAAGGCTGC), CDK1 (NM_001786.5; F:GCACCATATTTGCTGAACTAGC; R:GAGTGCCCAAAGCTCTGAAA), CCNB1 (NM_031966.4; R:AATAAGGCGAAGATCAACATGGC; F:TTTGTTACCAATGTCCCCAAGAG), CDKN2A (NM_000077.4; F:GCCGATCCAGGTCATGATGA; R:ACGGGTCGGGTGAGAGTG), and GAPDH (NM_002046.5; F:GAAGGTGAAGGTCGGAGTCAAC; R:AAGGGGTCATTGATGGCAAC). To visualize the results of the conventional PCR, the PCR products were analyzed through 3% agarose gel electrophoresis and visualized with ethidium bromide using a 50 bp DNA ladder.

### 4.6 Statistical Analysis

Statistical analysis was conducted using GraphPad Prism 8.0 software. All in vitro experiments were performed in triplicate, and the data are presented as mean ± standard deviation (SD). Comparisons between different groups and the control group were made using one-way analysis of variance (ANOVA) followed by Tukey’s multiple comparisons test. Statistical significance was defined as *p* < 0.05.

### 4.7 Data Acquisition, Processing, and Chemical Profiling

Chromatographic analysis was carried out using an ultraperformance liquid chromatography system coupled with a quadrupole time-of-flight tandem mass spectrometer (UHPLC-MS) (Xevo G2-XS, Waters Corp., Milford, USA), according Katchborian et al.^17^ Briefly, the separation was using C18 column (1.8 μm, 100 × 2.1 mm, ACQUITY UPLC HSS T3) maintained at 40 °C. The mobile phases consisted of (A) acidified water with 0.1% formic acid, and (B) pure acetonitrile, 1% of B in 0.1 min, 15% of B in 7.5 min, 8% of B in 8.5 min, 99% of B in 8.6 min, and 1% of B until 10 min. The flow rate was 0.5 mL/min.^17^ The electrospray ionization (ESI) source was operated in both positive and negative ionization modes to ensure broad analyte coverage. Data-independent acquisition (DIA) was performed using MS^E^, processed with MassLynx software (v4.2, Waters Corp., Milford, USA). To ensure analytical robustness, five independent analytical replicates were performed for CE. For other *Ocotea* spp., one UHPLC-MS injection was carried out per species.

The raw MS data files were first converted to .mzML format using Waters2mzML^73^ and subsequently imported into MZmine software (v4.3, MZmine Development Team) for data processing.^74^ Peaks were detected and processed through deconvolution, deisotoping, and alignment of identical peaks across chromatograms, followed by gap filling, duplication filtering, and subtraction of blank chromatograms. Features were then annotated based on their monoisotopic masses, with data from positive and negative ionization modes analyzed separately. Detailed parameters for the data processing workflow are provided in the Section S1 in the Supporting Information. The processed MS data were exported in.xlsx format. Annotation was conducted by screening monoisotopic masses with a specific genus database *Ocotea*DB^69,20^ and the broad database of the Dictionary of Natural Products.^75^ These annotations were classified as confidence level 3 and 2 according to the MSI: MSI confidence level-3 is MS^1^-based class assignment and MSI confidence level-2, MS2-based metabolite assignment^67,68^

### 4.8 Multivariate Statistical Analysis

Processed data, including peak area, *m*/*z*, and retention time, were imported into MetaboAnalyst 6.0 (Montreal, Canada, https://www.metaboanalyst.ca/) for multivariate statistical analysis (MSA). Before analysis, the data underwent sum normalization, log transformation, and Pareto scaling. An unsupervised principal component analysis (PCA) was initially performed, followed by heatmap analysis to visualize clustering patterns. Subsequently, orthogonal partial least-squares-discriminant analysis (OPLS-DA) was applied, using experimental cytotoxicity as the classification variable. To identify discriminant metabolites, variables important in projection (VIP) with values >3 and positive correlation coefficients with the activity were selected to differentiate active from inactive samples.^17,76,77^ The reliability of the OPLS-DA model was further assessed using 100 permutation tests. A metabolite was considered statistically significant only if log2 FC > 5. Overall, metabolite RT-*m*/*z* pairs were considered potential bioactive markers if they met the criteria: VIP > 3, log2 FC values >5 and positive CorrCoef.^17,49,52,56,76^

## Supplementary Material



## Notes

2D score plots of principal component analyses from UPLC-HRMS analysis and detailed parameters for the data processing workflow (PDF)

## Abbreviations:

CE: crude extract of *Ocotea villosa* leaf
5-FU: 5-fluorouracil
MSI: Metabolomic Standard Initiative.
