# Supplementary material for: Antiproliferative Activity Screening in Ocotea spp. Reveals Active Compounds in O. villosa with a Promising Mechanism of Action against Human Breast Cancer Cell Line MCF‑7
Source: ACS Omega. 2025 Dec 1;10(49):60585–602. doi: 10.1021/acsomega.5c08430 (PMC12713442; doi:10.1021/acsomega.5c08430)
Supplement: Supplementary file 1 [file ao5c08430_si_001.pdf]

Antiproliferative activity screening in *Ocotea* spp. reveals active compounds in *O. villosa* with a promising mechanism of action against human breast cancer cell line MCF-7

Fernanda Brito Leite Dornelas [1], Wanderleya Toledo dos Santos[1], Vanessa Viana Lempk[1], Juliana Leal Rodrigues da Costa[1], Clarissa Ferreira Cunha[2], Michael Murgu[3], Matheus Fernandes Alves[4], Albert Katchborian-Neto[4,5], José Otávio do Amaral Corrêa[1], Daniela Aparecida Chagas-Paula[4,6], Fernanda Maria Pinto Vilela[1], Ana Cláudia Chagas de Paula[1*].

[1]Department of Pharmaceutical Sciences. Federal University of Juiz de Fora, 36036-900, Juiz de Fora, MG, Brazil

[2]University Hospital, Federal University of Juiz de Fora, 36036-900, Juiz de Fora, MG, Brazil

[3] Waters Corporation, 06455-020, São Paulo, SP, Brazil

[4]Chemistry Institute. Federal University of Alfenas, 37130-001, Alfenas, MG, Brazil

[5]Center of Natural Sciences and Humanities, Federal University of ABC, Santo Andre, São Paulo, Brazil

[6] Department of Chemistry. Federal University of Juiz de Fora, 36036-900, Juiz de Fora, MG, Brazil

*Corresponding author

Ana Claudia Chagas de Paula Ladvocat, Department of Pharmaceutical Sciences.

Federal University of Juiz de Fora, Juiz de Fora, MG, Brazil.

Tel.: +55 32 2102-3803

Email address: ana.chagasdepaula@gmail.com

Supporting Information

Contents

Figure S1: 2D score plot of principal component analyses PC1 × PC2 (left) and PC1 x PC4 (right) from UPLC-HRMS analysis in ESI- mode………….. 2

Figure S2: 2D score plot of principal component analyses PC1 × PC2 (left) and PC1 x PC4 (right) from UPLC-HRMS analysis in ESI+ mode………….. 3

Section S1: Detailed parameters for the data processing workflow……….. 4

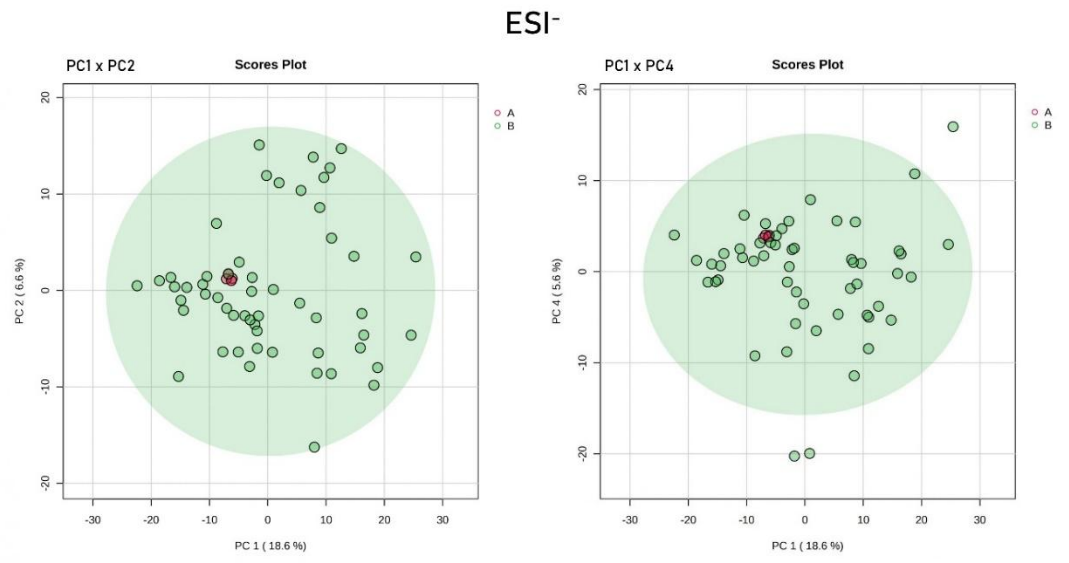

Figure S1: 2D score plot of principal component analyses PC1 × PC2 (left) and PC1 x PC4 (right) from UPLC-HRMS analysis in ESI− mode. Samples are color-coded as CE (red, 5 analytical replicates) and *Ocotea* spp. (green, single analytical data point per *Ocotea* species). Hotelling's T2 ellipses in the 2D plots represent the 95% confidence interval for *Ocotea* sp. PCA parameters: 8 components, R^2 = 0.521 (ESI−) and R^2 = 0.507 (ESI+).

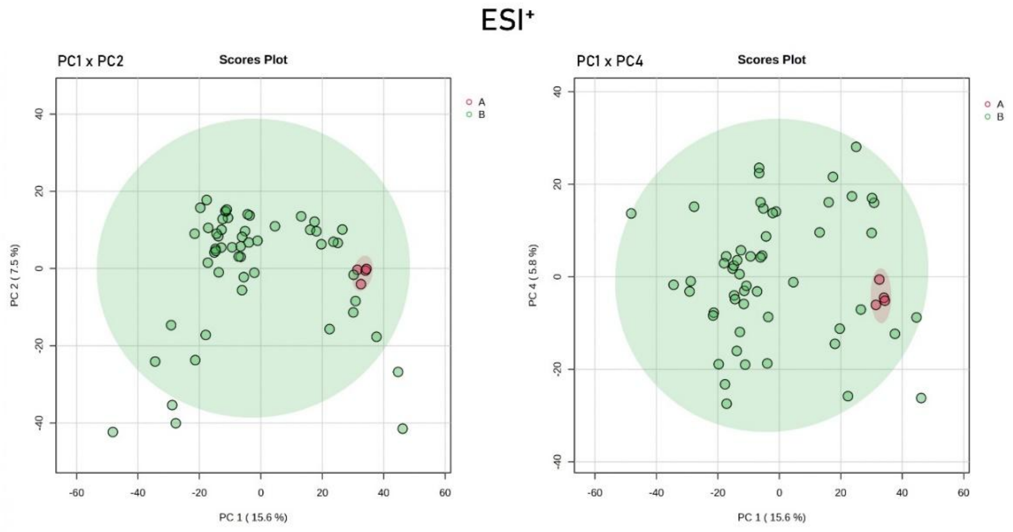

Figure S2: 2D score plot of principal component analyses PC1 × PC2 (left) and PC1 x PC4 (right) from UPLC-HRMS analysis in ESI+ mode. Samples are color-coded as CE (red, 5 analytical replicates) and *Ocotea* spp. (green, single analytical data point per *Ocotea* species). Hotelling's T2 ellipses in the 2D plots represent the 95% confidence interval for *Ocotea* sp. PCA parameters: 8 components, R^2 = 0.521 (ESI−) and R^2 = 0.507 (ESI+).

Section S1: Detailed parameters for the data processing workflow.

The raw data in ESI^+ were imported as .mzML files (e.g., B-01n.mzML; additional files B-02p, B-03n/p, B-04p, VI_AF-01p, VI_AF-02p, and VI_AF-03p), with vendor centroiding enabled and advanced import options disabled. No MS^1 *m/z* cropping was applied; denormalization of trap fragment scans was disabled; Mass detection was performed on all raw files for MS^1 and MS^2 scans using a centroid mass detector with a noise level of 200. Chromatogram building required ≥5 consecutive scans, minimum intensity per consecutive scan 5000, minimum absolute height 10000, and a scan-to-scan *m/z* tolerance of 0.005 *m/z* or 10 ppm (suffix "chromatograms"). Features were resolved with the Local Minimum Feature Resolver in the retention-time dimension (chromatographic threshold 0.85; minimum RT search range 0.05 min; minimum relative height 0.0; minimum peak-top/edge ratio 1.7; peak duration 0.0–1.5 min; ≥5 data points). MS/MS scan pairing was disabled; the $MS^1 \rightarrow MS^2$ precursor tolerance was 0.01 *m/z* or 10 ppm. Isotope grouping (^{13}C isotope filter; "deisotoped") was applied intra-sample with *m/z* tolerance 0.003 *m/z* or 5 ppm, RT tolerance 0.05 min, monotonic-shape filter off, maximum charge 2, and the most-intense representative isotope; features with MS^2 were not forcibly retained. Feature lists were aligned using the Join aligner (specific lists; output name "Aligned feature list_POS"). Where applicable downstream, tolerances were *m/z* 0.003 m/z or 5 ppm and RT 0.08 min. Library/spectral comparison parameters (applied where relevant) used spectral *m/z* tolerance 0.001 or 10 ppm at MS^2, a weighted cosine similarity (MassBank weights $mz^2 \cdot I$^0.5), minimum cosine 0.70, and "keep all and match to zero" handling for unmatched signals. Gap filling ("gap-filled") used intensity tolerance 0.20, *m/z* tolerance 0.003 *m/z* or 5 ppm, RT

tolerance 0.08 min, and a minimum of 4 data points. A duplicate-rows filter (suffix “filtered”) was then applied with NEW AVERAGE aggregation and the same *m/z* (0.003 *m/z* or 5 ppm), RT (0.08 min). Finally, blank/control subtraction was performed on the aligned feature list using specific blank files with a minimum of 3 detections in blanks; peak quantification was based on area, ratios were averaged, features below the specified fold-change threshold (FC = 3.0) were removed. All other unspecified parameters were set to default. An analogous set of parameters was applied to negative ionization (ESI^-), identical in all steps and tolerances except that the mass-detection noise threshold in ESI^- was set higher (500) to accommodate increased baseline noise. Processed data containing peak area, *m/z*-Rt pair was exported as a .csv table format for further data analysis.
